# Supplementary material for: HEART rate variability biofeedback for long COVID symptoms (HEARTLOC): protocol for a feasibility study
Source: BMJ Open. 2022 Nov 21;12(11):e066044. doi: 10.1136/bmjopen-2022-066044 (PMC9680157; doi:10.1136/bmjopen-2022-066044)
Supplement: Supplementary data [file bmjopen-2022-066044supp001.pdf]

Modified COVID-19 Yorkshire Rehabilitation Screening (C19-YRS)

Self-report version

Participant Identification Number:

HEARTLOC C19YRS form number:

Date: Time:

The purpose of this questionnaire is to find out more about your current problems following COVID-19 illness. Your responses will be recorded in your clinical notes. We will use this information to monitor your symptoms, offer treatments and assess response to treatment.

This questionnaire will take around 15 minutes. If there are any topics you don't want to talk about you can choose not to respond.

Do you consent for this information to be used for audit and research as well ? **Yes** ☐ **No** ☐

SYMPTOM SEVERITY

Please answer the questions below to the best of your knowledge.<br>*'Now' refers to how you feel now/this week (last 7 days).*<br>*"Pre-COVID" refers to how you were feeling prior to contracting the illness.*<br>*If you are unable to recall this, just state 'don't know'*<br><br>*Rate the severity of each problem on a scale of 0-3:*<br>***0 = None; no problem***<br>***1 = Mild problem; does not affect daily life***<br>***2 = Moderate problem; affects daily life to a certain extent***<br>***3 = Severe problem; affects all aspects of daily life; life-disturbing***			
1. Breathlessness	Breathlessness:	**Now**	**Pre-COVID**
	a) At rest	**0** ☐ **1** ☐ **2** ☐ **3** ☐	**0** ☐ **1** ☐ **2** ☐ **3** ☐
	b) Changing position e.g. from lying to sitting or sitting to lying	**0** ☐ **1** ☐ **2** ☐ **3** ☐	**0** ☐ **1** ☐ **2** ☐ **3** ☐
	c) On dressing yourself	**0** ☐ **1** ☐ **2** ☐ **3** ☐	**0** ☐ **1** ☐ **2** ☐ **3** ☐
	d) On walking up a flight of stairs	**0** ☐ **1** ☐ **2** ☐ **3** ☐	**0** ☐ **1** ☐ **2** ☐ **3** ☐
2. Cough/ throat sensitivity/ voice change	Cough/ throat sensitivity	**0** ☐ **1** ☐ **2** ☐ **3** ☐	**0** ☐ **1** ☐ **2** ☐ **3** ☐
	Change of voice	**0** ☐ **1** ☐ **2** ☐ **3** ☐	**0** ☐ **1** ☐ **2** ☐ **3** ☐
3. Fatigue (tiredness	Fatigue levels in your usual activities	**0** ☐ **1** ☐ **2** ☐ **3** ☐	**0** ☐ **1** ☐ **2** ☐ **3** ☐

not improved by rest)			
4. Smell/taste	Altered smell	**0** ☐ **1** ☐ **2** ☐ **3** ☐	**0** ☐ **1** ☐ **2** ☐ **3** ☐
	Altered taste	**0** ☐ **1** ☐ **2** ☐ **3** ☐	**0** ☐ **1** ☐ **2** ☐ **3** ☐
5. Pain/discomfort	Chest pain	**0** ☐ **1** ☐ **2** ☐ **3** ☐	**0** ☐ **1** ☐ **2** ☐ **3** ☐
	Joint pain	**0** ☐ **1** ☐ **2** ☐ **3** ☐	**0** ☐ **1** ☐ **2** ☐ **3** ☐
	Muscle pain	**0** ☐ **1** ☐ **2** ☐ **3** ☐	**0** ☐ **1** ☐ **2** ☐ **3** ☐
	Headache	**0** ☐ **1** ☐ **2** ☐ **3** ☐	**0** ☐ **1** ☐ **2** ☐ **3** ☐
	Abdominal pain	**0** ☐ **1** ☐ **2** ☐ **3** ☐	**0** ☐ **1** ☐ **2** ☐ **3** ☐
6. Cognition	Problems with concentration	**0** ☐ **1** ☐ **2** ☐ **3** ☐	**0** ☐ **1** ☐ **2** ☐ **3** ☐
	Problems with memory	**0** ☐ **1** ☐ **2** ☐ **3** ☐	**0** ☐ **1** ☐ **2** ☐ **3** ☐
	Problems with planning	**0** ☐ **1** ☐ **2** ☐ **3** ☐	**0** ☐ **1** ☐ **2** ☐ **3** ☐
7. Palpitations/ dizziness	Palpitations in certain positions, activity or at rest	**0** ☐ **1** ☐ **2** ☐ **3** ☐	**0** ☐ **1** ☐ **2** ☐ **3** ☐
	Dizziness in certain positions, activity or at rest	**0** ☐ **1** ☐ **2** ☐ **3** ☐	**0** ☐ **1** ☐ **2** ☐ **3** ☐
8. Post-exertional malaise (worsening of symptoms)	Crashing or relapse hours or days after physical, cognitive or emotional exertion	**0** ☐ **1** ☐ **2** ☐ **3** ☐	**0** ☐ **1** ☐ **2** ☐ **3** ☐
9. Anxiety/ mood	Feeling anxious	**0** ☐ **1** ☐ **2** ☐ **3** ☐	**0** ☐ **1** ☐ **2** ☐ **3** ☐
	Feeling depressed	**0** ☐ **1** ☐ **2** ☐ **3** ☐	**0** ☐ **1** ☐ **2** ☐ **3** ☐
	Having unwanted memories of your illness or time in hospital	**0** ☐ **1** ☐ **2** ☐ **3** ☐	**0** ☐ **1** ☐ **2** ☐ **3** ☐
	Having unpleasant dreams about your illness or time in hospital	**0** ☐ **1** ☐ **2** ☐ **3** ☐	**0** ☐ **1** ☐ **2** ☐ **3** ☐
	Trying to avoid thoughts or feelings about your illness or time in hospital	**0** ☐ **1** ☐ **2** ☐ **3** ☐	**0** ☐ **1** ☐ **2** ☐ **3** ☐
10. Sleep	Sleep problems, such as difficulty falling asleep, staying asleep or oversleeping	**0** ☐ **1** ☐ **2** ☐ **3** ☐	**0** ☐ **1** ☐ **2** ☐ **3** ☐

FUNCTIONAL ABILITY

11. Communication	Difficulty with communication/word finding difficulty/understanding others	**Now**	**Pre-COVID**
		**0** ☐ **1** ☐ **2** ☐ **3** ☐	**0** ☐ **1** ☐ **2** ☐ **3** ☐
12. Walking or moving around	Difficulties with walking or moving around	**0** ☐ **1** ☐ **2** ☐ **3** ☐	**0** ☐ **1** ☐ **2** ☐ **3** ☐
13. Personal care	Difficulties with personal tasks such as using the toilet or getting washed and dressed	**0** ☐ **1** ☐ **2** ☐ **3** ☐	**0** ☐ **1** ☐ **2** ☐ **3** ☐
14. Other activities of Daily Living	Difficulty doing wider activities, such as household work, leisure/sporting activities, paid/unpaid work, study or shopping	**0** ☐ **1** ☐ **2** ☐ **3** ☐	**0** ☐ **1** ☐ **2** ☐ **3** ☐
15. Social role	Problems with socialising/interacting with friends* or caring for dependants<br><br>*related to your illness and not due to social distancing/lockdown measures	**0** ☐ **1** ☐ **2** ☐ **3** ☐	**0** ☐ **1** ☐ **2** ☐ **3** ☐

OTHER SYMPTOMS

Please select any of the following symptoms you have experienced since your illness in the last 7 days.Please also select any previous problems that have worsened for you following your illness.

☐ Fever
☐ Skin rash/ discolouration of skin
☐ New allergy such as medication, food etc
☐ Hair loss
☐ Skin sensation (numbness/tingling/itching/nerve pain)
☐ Dry eyes/ redness of eyes
☐ Swelling of feet/ swelling of hands
☐ Easy bruising/ bleeding
☐ Visual changes
☐ Difficulty swallowing solids
☐ Difficulty swallowing liquids
☐ Balance problems or falls
☐ Weakness or movement problems or coordination problems in limbs
☐ Tinnitus
☐ Nausea
☐ Dry mouth/mouth ulcers
☐ Acid Reflux/heartburn
☐ Change in appetite
☐ Unintentional weight loss
☐ Unintentional weight gain
☐ Bladder frequency,urgency or incontinence
☐ Constipation, diarrhoea or bowel incontinence

☐ Change in menstrual cycles or flow
☐ Waking up at night gasping for air (also called sleep apnea)
☐ Thoughts about harming yourself

Other symptoms – free text

OVERALL HEALTH

How good or bad is your health overall in the last 7 days?

For this question, a score of 10 means the BEST health you can imagine. 0 means the WORST health you can imagine.

a) Now: WORST HEALTH 0 ☐ 1 ☐ 2 ☐ 3 ☐ 4 ☐ 5 ☐ 6 ☐ 7 ☐ 8 ☐ 9 ☐ 10 ☐ BEST HEALTH
b) Pre-Covid: WORST HEALTH 0 ☐ 1 ☐ 2 ☐ 3 ☐ 4 ☐ 5 ☐ 6 ☐ 7 ☐ 8 ☐ 9 ☐ 10 ☐ BEST HEALTH

EMPLOYMENT

Occupation: ______________________

Has your COVID-19 illness affected your work??

☐ No change
☐ On reduced working hours
☐ On sickness leave
☐ Changes made to role/ working arrangements (such as working from home or lighter duties)
☐ Had to retire/ change job
☐ Lost job

Any other comments/concerns:____

PARTNER/FAMILY/CARER PERSPECTIVE

This is space for your partner, family or carer to add anything from their perspective:

Corrado J, *et al. BMJ Open* 2022; 12:e066044. doi: 10.1136/bmjopen-2022-066044
