# Supplementary material for: HEART rate variability biofeedback for long COVID symptoms (HEARTLOC): protocol for a feasibility study
Source: BMJ Open. 2022 Nov 21;12(11):e066044. doi: 10.1136/bmjopen-2022-066044 (PMC9680157; doi:10.1136/bmjopen-2022-066044)
Supplement: Author's manuscript [file bmjopen-2022-066044.draft_revisions.pdf]

BMJ Open

BMJ Open is committed to open peer review. As part of this commitment we make the peer review history of every article we publish publicly available.

When an article is published we post the peer reviewers' comments and the authors' responses online. We also post the versions of the paper that were used during peer review. These are the versions that the peer review comments apply to.

The versions of the paper that follow are the versions that were submitted during the peer review process. They are not the versions of record or the final published versions. They should not be cited or distributed as the published version of this manuscript.

BMJ Open is an open access journal and the full, final, typeset and author-corrected version of record of the manuscript is available on our site with no access controls, subscription charges or pay-per-view fees (http://bmjopen.bmj.com).

If you have any questions on BMJ Open's open peer review process please email info.bmjopen@bmj.com

BMJ Open

# HEART rate variability biofeedback for LOng Covid symptoms (HEARTLOC): protocol for a feasibility study

| | |
|---|---|
| Journal: | *BMJ Open* |
| Manuscript ID | bmjopen-2022-066044 |
| Article Type: | Protocol |
| Date Submitted by the Author: | 28-Jun-2022 |
| Complete List of Authors: | Corrado, Joanna; University of Leeds, Academic Department of Rehabilitation Medicine; Leeds Teaching Hospitals NHS Trust<br>Halpin, Stephen; University of Leeds<br>Preston, Nick; University of Leeds<br>Whiteside, Diana; Leeds Community Healthcare NHS Trust<br>Tarrant, Rachel; Leeds Community Healthcare NHS Trust<br>Davison, Jennifer; Leeds Community Healthcare NHS Trust<br>Simms, Alexander; Leeds Teaching Hospitals NHS Trust<br>O'Connor, Rory J.; University of Leeds, Academic Department of Rehabilitation Medicine; Leeds Teaching Hospitals NHS Trust, National Demonstration Centre in Rehabilitation<br>Casson, Alexander; The University of Manchester<br>Sivan, Manoj; University of Leeds, Academic Department of Rehabilitation Medicine; Leeds Teaching Hospitals NHS Trust |
| Keywords: | COVID-19, Health informatics < BIOTECHNOLOGY & BIOINFORMATICS, REHABILITATION MEDICINE, CARDIOLOGY |
| | |

SCHOLARONE™
Manuscripts

# HEART rate variability biofeedback for LOng Covid symptoms (HEARTLOC): protocol for a feasibility study

**Corrado J[1,2,3], Halpin SJ[1,2,3], Preston N[1], Whiteside D[2], Tarrant R[2], Davison J[2], Simms AD[2,4], O'Connor RJ[1,3], Casson AJ[1,4,5], Sivan M[1,2,3]**

*Affiliations:*

[1] Academic Department of Rehabilitation Medicine, Leeds Institute of Rheumatic and Musculoskeletal Medicine, University of Leeds

[2] Covid rehabilitation service, Leeds Community Healthcare NHS Trust

[3] National Demonstration Centre of Rehabilitation Medicine, Leeds Teaching Hospitals NHS Trust

[4] Department of Cardiology, Leeds Teaching Hospitals NHS Trust

[5] Department of Electrical and Electronic Engineering, University of Manchester

[4] Manchester the Henry Royce Institute, Manchester, United Kingdom

**Correspondence:**

Dr Manoj Sivan MD FRCP Ed

Academic Department of Rehabilitation Medicine

Leeds Institute of Rheumatic and Musculoskeletal Medicine

University of Leeds

Martin Wing, Leeds General Infirmary, LS1 3EX

Tel: 0044 1133922564

E-mail address: m.sivan@leeds.ac.uk

**Word count:** 2930 (excluding abstract and references)

For peer review only

# Abstract (277/300)

## Introduction

Long covid (LC), also known as Post-COVID-19 syndrome, refers to symptoms persisting 12 weeks after COVID-19 infection. It affects up to 1 in 7 people contracting the illness and causes a wide range of symptoms, including fatigue, breathlessness, palpitations, dizziness, pain and brain fog. Many of these symptoms can be linked to dysautonomia or dysregulation of the autonomic nervous system after SARS-CoV2 infection. This study aims to test the feasibility and estimate the efficacy, of the Heart Rate Variability Biofeedback (HRV-B) technique via a standardised slow diaphragmatic breathing programme in individuals with LC.

## Methods and Analysis

30 adult LC patients with symptoms of palpitations or dizziness and an abnormal NASA Lean Test (NLT) will be selected from a specialist Long COVID rehabilitation service. They will undergo a 4-week HRV-B intervention using a Polar chest strap device linked to the Elite HRV phone application while undertaking the breathing exercise technique for two 10-min periods every day for at least 5 days a week. Quantitative data will be gathered during the study period using: HRV data from the chest strap and wrist-worn Fitbit, the modified COVID-19 Yorkshire Rehabilitation Scale (C19-YRSm), composite autonomic symptom score (COMPASS 31), World Health Organisation Disability Assessment Schedule (WHODAS 2.0) and EQ-5D-5L health related quality of life measures. Qualitative feedback on user experience and feasibility of using the technology in a home setting will also be gathered. Standard statistical tests for correlation and significant difference will be used to analyse the quantitate data.

## Ethics and Dissemination

The study has received ethical approval from Health Research Authority (HRA) Leicester South Research Ethics Committee (21/EM/0271). Dissemination plans include academic and lay publications.


For peer review only

## Study Registration

Clinicaltrials.gov No: NCT05228665


## Keywords

Post-COVID-19 condition, post-COVID-19 syndrome, dysautonomia, autonomic dysfunction, sympathetic, parasympathetic, rehabilitation, technology


# Article summary

### Strengths and limitations of the study

- To our knowledge, this is the first study of HRVB in long covid and will provide new information regarding the feasibility of the technology-based intervention in this condition.
- The estimation of efficacy will determine the scope and sample size for a larger controlled trial in the condition that currently has no definitive treatments
- The study will provide preliminary evidence on the correlation between long covid symptoms and dysautonomia.
- The limitation of this study is the small sample size of 30 participants which might not give an accurate estimate of efficacy.
- HRV-B is a technology-based intervention, therefore its take-up could be limited in those with a lack of experience in using digital technology in daily life, particularly those from less privileged backgrounds.

# Introduction

Post-COVID-19 syndrome or Long covid (LC) refers to persistent symptoms 12 weeks after SARS-COV2 infection and can include symptoms of physical fatigue, cognitive fatigue or “brain fog”, breathlessness, pain and psychological distress.[1 2] An estimated 1.4 million people are reported to be affected by LC in the UK alone.[3] The condition can be highly debilitating for some, particularly middle-aged individuals who were previously functioning at

For peer review only

a high level and in demanding vocational roles.[4] Many will experience significant disruption to employment, social and caregiving roles, and participation in society.

Many LC symptoms such as palpitations, dizziness, fatigue, pain and breathlessness can be explained by the theory of dysautonomia.[5 6] This is a state of episodic dysregulation in the autonomic nervous system (ANS) with sympathetic overdrive and reduced parasympathetic activity. Dysautonomia plays a significant role in the symptomology of many long-term conditions including multiple sclerosis, Parkinson's disease, diabetes mellitus, fibromyalgia, chronic fatigue syndrome and migraine.[7]

One way of estimating and measuring autonomic function is through Heart Rate Variability (HRV), as cardiac rate and rhythm are controlled largely by the autonomic nervous system. The parasympathetic nervous system chiefly activates a slowing of heart rate through the vagus nerve, and the sympathetic response acts through the activation of β-adrenergic receptors.[8] HRV can be measured either in the time domain or frequency domain. HRV represents a measure of the variation in time between heartbeats (captured on an ECG strip as a time interval between the R waves of the QRS complexes). A low HRV is associated with sympathetic nervous system activation, also described as a state of 'fight or flight'. Higher HRV correspond with parasympathetic nervous system activation and is believed to reflect a state of rest and recovery. Lower HRV has been observed to be associated with fatigue and pain symptoms of chronic fatigue syndrome/myalgic encephalomyelitis (ME/CFS) and fibromyalgia [9-11], as well as other chronic physical and mental health pathologies including asthma, anxiety and stress.[9-13]

## Heart rate variability biofeedback (HRV-B)

When physiological parameters such as HRV are monitored in real-time with self-regulation techniques such as breathing exercises applied to influence the parameters, this is known as biofeedback (BFB).[14 15] In this study, for monitoring and modulating the HRV, we are utilising breathing techniques to encourage the predominance of parasympathetic nervous activity through vagus nerve activation. To the best of our knowledge, there have not yet been any studies of HRV-B in LC. However, HRV-B using breathing techniques has been tested in other

For peer review only

clinical conditions such as asthma[12], depression[16] and fibromyalgia[11]. A normal respiratory rate is between 12 and 20 breaths per minute.[17] The optimal breathing frequency to produce maximal increase in HRV varies for each individual but on average is between 5.5 and 6 breaths per minute and is known as resonant breathing.[12 17 18] Resonant breathing helps to restore autonomic balance due to baroreflex gain and vagal activation. [12 17-19]

There are several means of assessing HRV but most commonly these include the use of either wearable devices such as smartwatches or chest straps, or through small attachable Holter ECG units. These are non-invasive and readily available, although reliability differs between devices and platforms. Many commercial HRV devices are associated with smartphone app technology which can be readily downloaded and made available to participants for monitoring. Of the consumer grade devices available to monitor HRV the Polar H10 chest strap is felt to be the most reliable and remains accurate even during high-intensity activity.[20] The Polar H10 can be linked with the Elite HRV app which provides real time feedback on HRV and the user's response to breathing techniques. The combination of Polar H10 chest strap and Elite HRV app has been effectively used to harness real time physiological data, for example in athletes.[21] In contrast, many wrist worn devices such as Fitbit return a measure of HRV only while the user is asleep due to motion and other interference sources, meaning real-time HRV-B is not possible.

The aim of this study is to determine the feasibility and impact of a structured HRV-B regime incorporating diaphragmatic breathing exercise, on LC symptoms. We wish to test the acceptability and compliance of the intervention and estimate effect on symptoms using standardised validated measures of LC and dysautonomia.

## Aims and objectives

The aim of this study is: To assess the feasibility of a 4-week HRV biofeedback structured breathing programme in individuals with LC.

The objectives include:

1. Does breathing exercises through HRV-B increase HRV amongst participants with LC?

For peer review only

2. Are consumer grade monitors appropriate technology to use for HRV-B in the domiciliary setting?
3. Does regular HRV-B have any effect on LC symptoms?

# Methods

## Study design

This is a phase 2 uncontrolled open-label feasibility study of a home technology-based HRV-B in 30 individuals with LC. Potential participants will be identified through the Leeds COVID-19 Rehabilitation Service, based at Leeds Community Healthcare NHS Trust. The study period will be 6 weeks for each participant.

## Eligibility criteria

The inclusion criteria are

- Age > or = 18 years
- Confirmed LC diagnosis as per the NICE criteria for post-COVID syndrome [1]
- Self-rating of at least 'moderate' or 'severe' on dysautonomia questions of palpitations or dizziness on the C19-YRSm [22]; and
- Abnormal NASA Lean Test (NLT)[23-25]
    - HR increase of 30bpm or ≥120bpm
    
      or
    - BP decrease of 20mmHg systolic or 10 mmHg diastolic in the first 3 minutes of standing

NLT is an accepted measure of cardiovascular instability and is conducted at initial assessment clinic for all LC service users in the Leeds COVID rehabilitation service. The patient lies down for 2 to five minutes prior to the test with HR and BP taken each minute to calculate average supine values. They then stand with heels 6 inches from a wall and lean back against it with HR and BP taken each minute for 10 min. Abnormal results (as described above) are demonstrated through orthostatic hypotension or tachycardia on standing which are hallmarks of dysautonomia and therefore objectively quantifiable. The participants who have dysautonomia symptoms but do not meet the mentioned thresholds will not be



For peer review only

included in this feasibility study but will be potential recruits for future larger scale studies using the same intervention.

The exclusion criteria are

- Unable to use the wearable or smartphone app technology
- Cognitive difficulties or mental health disorders causing inability to consent
- Any cardiac arrhythmias that are being planned for further investigations and specialist management in the Cardiology service
- Any unstable cardiorespiratory disease which needs further medical interventions (except asthma management)

## Equipment and Technology

To collect medium-term HRV data, participants will wear a Fitbit Charge 5 smartwatch for a total of 6 weeks. The HRV-B itself will be conducted using a Polar H10 chest strap for 10 minutes twice daily. This connects via Bluetooth to the Elite HRV smartphone app which is downloaded to participants' phones. Participants will aim to increase their HRV score as displayed in Elite HRV in real time using a diaphragmatic breathing technique (Figure 1). Omron M2 blood pressure monitor (endorsed by the British Hypertension Society) will be used to conduct NASA Lean test (NLT) in clinic and the adapted Autonomic Profile (aAP)[26].

Insert Fig 1 about here

## Study phases

The study will be carried out in the following three phases:

- Pre HRV-B phase
- HRV-B phase
- Post HRV-B phase

### Pre HRV-B phase

The participant will either be invited to a research clinic or visited at their home by a member of the research team (first appointment A1). They would have already received the participant information sheet (PIS) at screening and would have had more than 24 hours to read and understand the content of the PIS. Written consent will be signed by the participant

For peer review only

and the researcher during this first visit. Devices and baseline outcome measures used in this stage are:

- Fitbit charge 5 device and the Fitbit smartphone application: The participant will be requested to have the Fitbit device on most of the time during the 6-week period. The application records HRV at night along with other measures of sleep (sleep stages, HR) and daytime activity (such as step count).
- Adapted Autonomic Profile (aAP): This is an autonomic profile test developed by St Mary's Hospital and the National Hospital for Neurology and Neurosurgery and later adapted for domiciliary use during the COVID-19 pandemic.[26] Participants are asked to monitor their heart rate and blood pressure on lying, and at 3 minutes of standing at various intervals over 24 hours, including after waking, after eating breakfast/ lunch/ dinner, before and after 5 minutes of exercise, and before bed (Supplementary file 1). Abnormal results are calculated using the same criteria for heart rate and blood pressure differences as the NLT (HR increase > 30/min or BP drop >20 mm Hg).

The A1 appointment will last approximately 2 hours and may be longer for those with cognitive fatigue or 'brain fog'. If felt necessary, it will be divided into two one-hour visits to reduce cognitive fatigue.

### HRV-B phase

One week after the A1 appointment, the participant will be either invited to attend a research clinic or visited at home by a researcher (second appointment A2) to commence the HRV-B study phase. This involves:

- Polar H10 chest strap and Elite HRVB application: The participant will be familiarised with the technology and introduced to a paced breathing regimen via a one-to-one demonstration. They will be instructed to perform the breathing technique using the application at least twice a day, 10 minutes per session, for a period of 4 weeks. The chest strap device will record HRV for the duration of the session, and the data gets recorded in the application. Whilst this phase is ongoing, participants will continue to wear the Fitbit Charge 5 device for the duration of this phase.
- C19-YRSm: The COVID-19 Yorkshire Rehabilitation Scale (C19YRS) is the literature's first condition-specific patient recorded outcome measure which has been validated in the LC population.[27 28] The modified scale provides a symptom severity score (out

For peer review only

of 30), functional disability score (out of 15), other symptoms score (out of 25) and overall health score (out of 10).[29] The participant will complete C19YRSm (Supplementary file 2) at weekly intervals to monitor the impact of the intervention on LC symptoms. They will also have weekly telephone reviews with study researchers for troubleshooting and to ensure maximal compliance with the study.

### Post HRV-B phase

The participant will be asked to stop the HRV-B intervention after completing 4 weeks of the treatment. They will be asked to continue using the Fitbit device for another week when not doing the intervention. They will then either be invited to a research clinic or be visited at home by a study researcher. At this appointment (A3), the participant will complete:

- COMPASS (Composite Autonomic Symptom Score): The COMPASS 31 will be completed by the participant at the initial visit and again 6 weeks later at the end of the study. Autonomic symptoms are scored for different domains including orthostatic intolerance, vasomotor, secretomotor, gastrointestinal, bladder and pupillomotor. Total scores for each domain are multiplied by a set weighting and then added together to provide a score out of 100 representing severity of autonomic symptoms. A higher score represents greater severity.[30]
- C19 YRSm The C19-YRSm will be completed by the patient every week for a total of 6 weeks. There will be a total of 7 C19-YRS documents completed.
- WHODAS: This is validated generic measure of functioning and disability. The 36-item scale captures six domains of life (cognition, mobility, self-care, getting along, life activities and participation) with a summary score ranging from 0 (no disability) to 100 (full disability)[31 32]
- EQ-5D-5L: The EQ-5D-5L instrument, provided by the EuroQol Group, is one of widely used quality of life measures, consists of five items covering: mobility, self-care, usual activities, pain/discomfort, and anxiety/depression.[33] The item scores can be converted into a total index score by applying health preference weights elicited from a general population. This index score can also be used in economic evaluations to assess the cost-effectiveness of health interventions.[34]

During the A3 appointment, the Polar H10 strap and the Fitbit device will be retrieved.

For peer review only

The participants will be invited to complete a further C19-YRS, by email or postal four weeks after completion aAP for 24 hours and to email or post the results to the study researcher.

## Outcome measures

**The primary outcome measure** is the C19YRSm, a self-reported patient-reported outcome measure to assess LC symptom severity, functional disability, and overall health status.

**Secondary outcome measures** include:

***Heart rate measures from chest strap:***

- 7-day average HRV score out of 100 - quantified by the Elite HRV app via the root mean square of successive differences between normal heartbeats (rMSSD). A natural log (ln) is applied to this figure and then expanded to generate a 1 to 100 score
- Mean R-R interval
- Heart rate
- rMSSD
- SDNN (standard deviation of NN intervals)
- Total Power
- Low frequency power (LF)
- High frequency power (HF)
- LF:HF ratio

***Fitbit Data:***

- Sleep staging data
- Resting heart rate
- Daily activity levels e.g. step count and exercise type and duration

***Patient Reported Outcome Measures:***

- Composite Autonomic Symptom Score (COMPASS 31)
- World Health Organisation Disability Assessment Schedule (WHODAS)
- EQ5D health related quality of life assessment (EQ-5D-5L)
- NASA Lean Test (NLT) – heart rate and blood pressure data
- adapted Autonomic Profile (aAP) – heart rate and blood pressure data

We will also ask participants about the feasibility and acceptability of HRVB as a management strategy for LC.

For peer review only

A summary of the schedule for the completion of outcome measures is shown in Table 1.

Insert Table 1 here

Table 1. Outcome measures summary schedule

| | Initial assessment Clinic | Pre HRV-B phase (1 week) | HRV-B phase (4 weeks) | Post HRV-B phase (1 week) |
|---|---|---|---|---|
| Autonomic screening NLT | √ | | | √ |
| Autonomic function COMPASS 31 | | √ | | √ |
| Home autonomic test aAP | √ | | | √ |
| Fitbit wrist strap HRV, sleep data | | √ | √ daily | √ |
| Polar H10 chest strap HRV data | | | √ daily | |
| LC specific PROM C19-YRSm | | √ | √ weekly | √ |
| Daily function | | √ | | √ |
| Quality of life | | √ | | √ |

## Statistical Analysis

Quantitative data from standardised questionnaires will be scored as per standard procedures. Data downloaded from the wearable devices will be extracted, cleaned, and summarised using specific software packages, including Matlab and Python. Quantitative data will be analysed with simple descriptive statistics. The presence and magnitude of pre and post-intervention differences will be examined using repeated paired-sample T-tests (with Bonferroni adjustment for multiple comparisons), and the effect size will be explored using both ANOVA partial Eta squared, and Cohen's d. Additional exploratory analyses may also be performed to fully analyse the dataset produced, guided by the findings of the descriptive statistics.

For peer review only

## Patient and public involvement

Members of the patient advisory group with lived experience of long covid have been involved in the design, development, and delivery of the project. Members of the patient advisory group attended proposal research planning meetings and shared their experiences on symptoms of dysautonomia which helped shaped the research question, design and outcome measures of this study. Members of this group have contacts with wider patient community groups and helped disseminate information about the study. The advisory group meets quarterly with the research team to review progress, ensure the research continues to answer relevant issues and that findings can inform long covid care. The group will be involved in the dissemination of research findings and writing lay summary reports that will be shared with the participants.

## Ethics and dissemination

The study has received ethical approval from Health Research Authority (HRA) Leicester South Research Ethics Committee (21/EM/0271). Informed consent will be obtained from all participants. Potential participants will have a minimum of 24 hours to review the PIS and discuss queries with the researcher prior to signing the written consent. GDPR rules will be strictly followed for all data gathered during the study. All data will be fully anonymised as soon as practical. All devices used are CE marked and are being used for their intended purposes. There is potential for minor skin irritation from wearing the Fitbit and Polar H10 devices. This will be enquired about at each weekly telephone review.

For participants with cognitive fatigue or 'brain fog' relating to LC, the length of the appointments with the researcher (A1, A2, A3) may be longer than normal. Supplementary written information will be provided, and if necessary, each of these appointments may be conducted in two shorter sessions to reduce information overload and possible impact on LC symptoms. Participants will be advised that they do not need to proceed with the

For peer review only

appointments or the study if they do not want to. All appointments other than the initial NLT can occur at the participants' homes to reduce travel and inconvenience. Participants are free to withdraw at any point in the study. They will be encouraged to give reasons for the withdrawal, but it will not be compulsory to give a reason for withdrawal.

Dissemination will include both academic publications and lay summaries in various formats. Academic outputs will include both medical and engineering literature. Policy impact will be aided by our strong existing links to NHS England and the UK Long COVID National Task Force. Dr Sivan, who leads the NHR project Long Covid Multidisciplinary consortium for Optimising Treatments and Services across the NHS (LOCOMOTION)[35], is also advisor for the World Health Organisation (WHO - Europe) on COVID-19 rehabilitation and is also involved in the WHO working party developing a core set of outcome measures for LC.

## Conflicts of interest

Manoj Sivan is an advisor to the World Health Organisation (WHO) for the Long COVID policy in Europe.

## Acknowledgements

The authors would like to thank individuals with autonomic problems in long covid and healthcare professionals from the Leeds Covid Rehabilitation service who provided valuable suggestions and feedback during the iterative process of development of this protocol. We are grateful to the Patient Advisory Group for its involvement in all stages of this study.

## Funding statement

This research is supported by IAA EPSRC [Ref 112538] with University of Leeds as the sponsor organisation and the Leeds Community Healthcare NHS Trust Covid Rehabilitation service as the research site organisation.

## Data statement

We will use Open Science Framework (OSF) to share of all research outputs, including data, codes, and other types of information that has the potential to aid the advancement of scientific progress and benefit other researchers by adding transparency to the research process. Data will also be shared via the University of Leeds's public data repository to increase exposure. The OSF will consist of two levels: a data dictionary with basic info about the study, and a more detailed dataset (e.g., for further analysis/meta-analysis). Data will be issued with a Digital Object Identifier (DOI) which will allow it to be referenced and make it easier for others to identify and access relevant files.

For peer review only

## Supplementary files

1 adapted Autonomic Profile (aAP) diary and instruction sheet
2 Modified Covid Yorkshire Rehabilitation Scale (C19-YRSm) questionnaire

## Author contributions

MS and AC conceptualised the study. MS, AC and RJOC were awarded EPSRC IAA pump-priming grant for the feasibility study with MS as the Principal Investigator. All authors contributed to the study design and obtained ethical approval. JC wrote an initial draft of the paper by adapting the grant proposal and the ethics protocol. All authors approved the final manuscript. All authors will contribute to recruitment, data acquisition and analysis of the study findings. MS is the corresponding author and guarantor.

## Figure legends

Fig 1. Heart Rate Variability Biofeedback (HRV-B) using a breathing technique and chest strap for real time HRV monitoring. Polar H10 picture from Wikimedia commons, reprinted under CC BY-SA 3.0 license. EliteHRV screenshot from Wikimedia commons, reprinted under CC BY-SA 4.0 license.

For peer review only

# References


1. National Institute for Health and Care Excellence (NICE) Scottish Intercollegiate Guidelines Network (SIGN) and Royal College of General Practitioners (RCGP). COVID-19 rapid guideline: managing the long- term effects of COVID-19. London: NICE 2022.
2. Crook H, Raza S, Nowell J, et al. Long covid—mechanisms, risk factors, and management. *bmj* 2021;374
3. Office of National Statistics. Prevalence of ongoing symptoms following coronavirus (COVID-19) infection in the UK: 1 June 2022. London: ONS 2022.
4. Statistics OoN. Coronavirus and the social impacts of 'long COVID' on people's lives in Great Britain: 7 April to 13 June 2021. 2021; https://www.ons.gov.uk/peoplepopulationandcommunity/healthandsocialcare/conditionsanddiseases/articles/coronavirusandthesocialimpactsoflongcovidonpeopleslivesingreatbritain/7aprilto13june2021. (accessed 02.05.2022).
5. Dani M, Dirksen A, Taraborrelli P, et al. Autonomic dysfunction in 'long COVID': rationale, physiology and management strategies. *Clin Med (Lond)* 2021;21(1):e63-e67. doi: 10.7861/clinmed.2020-0896 [published Online First: 2020/11/28]
6. Shouman K, Vanichkachorn G, Cheshire WP, et al. Autonomic dysfunction following COVID-19 infection: an early experience. *Clin Auton Res* 2021;31(3):385-94. doi: 10.1007/s10286-021-00803-8 [published Online First: 2021/04/17]

7. Zalewski P, Slomko J, Zawadka-Kunikowska M. Autonomic dysfunction and chronic disease. *Br Med Bull* 2018;128(1):61-74. doi: 10.1093/bmb/ldy036 [published Online First: 2018/11/13]

8. Heart rate variability. Standards of measurement, physiological interpretation, and clinical use. Task Force of the European Society of Cardiology and the North American Society of Pacing and Electrophysiology. *Eur Heart J* 1996;17(3):354-81. [published Online First: 1996/03/01]

9. Fournie C, Chouchou F, Dalleau G, et al. Heart rate variability biofeedback in chronic disease management: A systematic review. *Complement Ther Med* 2021;60:102750. doi: 10.1016/j.ctim.2021.102750 [published Online First: 2021/06/13]

10. Escorihuela RM, Capdevila L, Castro JR, et al. Reduced heart rate variability predicts fatigue severity in individuals with chronic fatigue syndrome/myalgic encephalomyelitis. *J Transl Med* 2020;18(1):4. doi: 10.1186/s12967-019-02184-z [published Online First: 2020/01/08]

11. Hassett AL, Radvanski DC, Vaschillo EG, et al. A pilot study of the efficacy of heart rate variability (HRV) biofeedback in patients with fibromyalgia. *Appl Psychophysiol Biofeedback* 2007;32(1):1-10. doi: 10.1007/s10484-006-9028-0 [published Online First: 2007/01/16]

12. Lehrer PM, Vaschillo E, Vaschillo B, et al. Biofeedback treatment for asthma. *Chest* 2004;126(2):352-61. doi: 10.1378/chest.126.2.352 [published Online First: 2004/08/11]

13. Goessl VC, Curtiss JE, Hofmann SG. The effect of heart rate variability biofeedback training on stress and anxiety: a meta-analysis. *Psychol Med* 2017;47(15):2578-86. doi: 10.1017/S0033291717001003 [published Online First: 2017/05/10]

14. Lehrer P, Vaschillo B, Zucker T, et al. Protocol for heart rate variability biofeedback training. *Biofeedback* 2013;41(3):98-109. doi: https://doi.org/10.5298/1081-5937-41.3.08

15. Gevirtz R. The promise of heart rate variability biofeedback: Evidence-based application. *Biofeedback* 2013;41(3):110-20. doi: https://doi.org/10.5298/1081-5937-41.3.01

16. Karavidas MK, Lehrer PM, Vaschillo E, et al. Preliminary results of an open label study of heart rate variability biofeedback for the treatment of major depression. *Appl*

For peer review only

*Psychophysiol Biofeedback* 2007;32(1):19-30. doi: 10.1007/s10484-006-9029-z [published Online First: 2007/03/03]

17. Vaschillo EG, Vaschillo B, Lehrer PM. Characteristics of resonance in heart rate variability stimulated by biofeedback. *Appl Psychophysiol Biofeedback* 2006;31(2):129-42. doi: 10.1007/s10484-006-9009-3 [published Online First: 2006/07/14]
18. Lehrer PM, Gevirtz R. Heart rate variability biofeedback: how and why does it work? *Front Psychol* 2014;5:756. doi: 10.3389/fpsyg.2014.00756 [published Online First: 2014/08/08]
19. Pagaduan JC, Chen YS, Fell JW, et al. Can Heart Rate Variability Biofeedback Improve Athletic Performance? A Systematic Review. *J Hum Kinet* 2020;73:103-14. doi: 10.2478/hukin-2020-0004 [published Online First: 2020/08/11]
20. Gilgen-Ammann R, Schweizer T, Wyss T. RR interval signal quality of a heart rate monitor and an ECG Holter at rest and during exercise. *Eur J Appl Physiol* 2019;119(7):1525-32. doi: 10.1007/s00421-019-04142-5 [published Online First: 2019/04/21]
21. Flatt AA, Howells D. Effects of varying training load on heart rate variability and running performance among an Olympic rugby sevens team. *J Sci Med Sport* 2019;22(2):222-26. doi: 10.1016/j.jsams.2018.07.014 [published Online First: 2018/07/30]
22. Sivan M, Preston N, Parkin A, et al. The modified COVID-19 Yorkshire Rehabilitation Scale (C19-YRSm) patient-reported outcome measure for Long Covid or Post-COVID-19 syndrome. *J Med Virol* 2022 doi: 10.1002/jmv.27878 [published Online First: 2022/05/24]
23. Bungo MW, Charles JB, Johnson PC, Jr. Cardiovascular deconditioning during space flight and the use of saline as a countermeasure to orthostatic intolerance. *Aviat Space Environ Med* 1985;56(10):985-90. [published Online First: 1985/10/01]
24. Lee J, Vernon SD, Jeys P, et al. Hemodynamics during the 10-minute NASA Lean Test: evidence of circulatory decompensation in a subset of ME/CFS patients. *J Transl Med* 2020;18(1):314. doi: 10.1186/s12967-020-02481-y [published Online First: 2020/08/18]
25. Hyatt KH, Jacobson LB, Schneider VS. Comparison of 70 degrees tilt, LBNP, and passive standing as measrues of orthostatic tolerance. *Aviat Space Environ Med* 1975;46(6):801-8. [published Online First: 1975/06/01]

For peer review only

26. Sivan M, Corrado J, Mathias C. The Adapted Autonomic Profile (Aap) Home-Based Test for the Evaluation of Neuro-Cardiovascular Autonomic Dysfunction. . *Preprints* 2022 doi: 10.20944/preprints202206.0325.v2

27. Sivan M, Halpin S, Gee J, et al. The self-report version and digital format of the COVID-19 Yorkshire Rehabilitation Scale (C19-YRS) for Long Covid or Post-COVID syndrome assessment and monitoring. *Advances in CLinical Neuroscience and Rehabilitation* 2021;20(3)

28. O'Connor RJ, Preston N, Parkin A, et al. The COVID-19 Yorkshire Rehabilitation Scale (C19-YRS): application and psychometric analysis in a post-COVID-19 syndrome cohort. *Journal of Medical Virology* 2022;94(3):1027-34.

29. Sivan M, Preston NJ, Parkin A, et al. The modified COVID-19 Yorkshire Rehabilitation Scale (C19-YRSm) patient-reported outcome measure for Long Covid or Post-COVID syndrome. *medRxiv* 2022:2022.03.24.22272892. doi: 10.1101/2022.03.24.22272892

30. Sletten DM, Suarez GA, Low PA, et al. COMPASS 31: a refined and abbreviated Composite Autonomic Symptom Score. *Mayo Clin Proc* 2012;87(12):1196-201. doi: 10.1016/j.mayocp.2012.10.013 [published Online First: 2012/12/12]

31. Garin O, Ayuso-Mateos JL, Almansa J, et al. Validation of the "World Health Organization Disability Assessment Schedule, WHODAS-2" in patients with chronic diseases. *Health Qual Life Outcomes* 2010;8:51. doi: 10.1186/1477-7525-8-51 [published Online First: 2010/05/21]

32. Ustun TB, Kostanjsek N, Chatterji S, et al. Measuring health and disability : manual for WHO Disability Assessment Schedule (WHODAS 2.0). 2010. https://www.who.int/publications/i/item/measuring-health-and-disability-manual-for-who-disability-assessment-schedule-(-whodas-2.0) (accessed 19.06.2022).

33. Herdman M, Gudex C, Lloyd A, et al. Development and preliminary testing of the new five-level version of EQ-5D (EQ-5D-5L). *Qual Life Res* 2011;20(10):1727-36. doi: 10.1007/s11136-011-9903-x [published Online First: 2011/04/12]

34. Brooks R. EuroQol: the current state of play. *Health Policy* 1996;37(1):53-72. doi: 10.1016/0168-8510(96)00822-6 [published Online First: 1996/06/06]

35. Sivan M, Greenhalgh T, Darbyshire JL, et al. LOng COvid Multidisciplinary consortium Optimising Treatments and servIces acrOss the NHS (LOCOMOTION): protocol for a


For peer review only

mixed-methods study in the UK. *BMJ Open* 2022;12(5):e063505. doi: 10.1136/bmjopen-2022-063505 [published Online First: 2022/05/18]

For peer review only

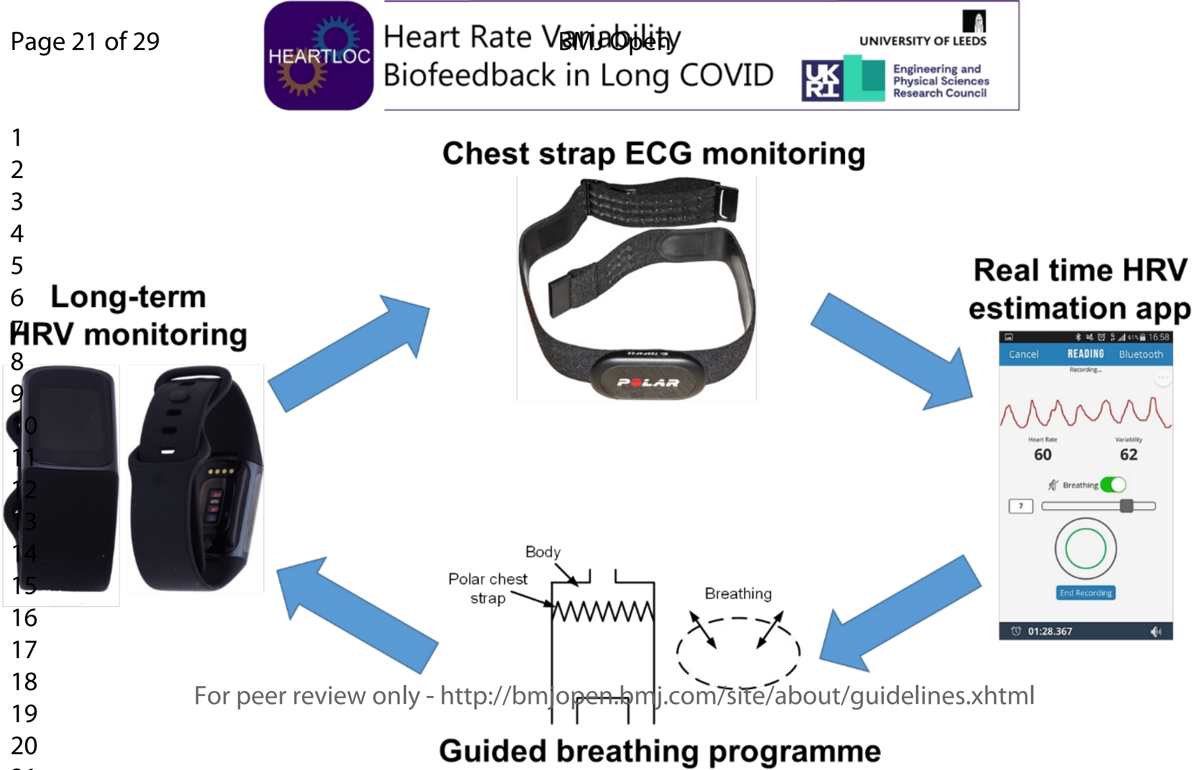


For peer review only - http://bmjopen.bmj.com/site/about/guidelines.xhtml

# The aAP diary sheet

**Participant Initials** **Date**

***Food or fluid intake** – please state what food or drink, including alcohol, was consumed
***Activity** (can be physical, cognitive or emotional) – please state what was the activity and for how long

| Enter time | Position/Activity | Blood Pressure | Heart Rate | Symptoms | Other details |
|---|---|---|---|---|---|
| **EARLY MORNING (ON WAKING)** Time: _ _ hr _ _min | | | | | |
| _ _ hr _ _ min | Lying | _ _ _/ _ _ _ sys. diast | | | |
| _ _ hr _ _min | After 3 min sitting | | | | |
| _ _ hr _ _min | After 3 min standing | | | | |
| **BREAKFAST** Time: _ _ hr _ _min; Details of food/fluid*: | | | | | |
| _ _ hr _ _ min | Lying | | | | |
| _ _ hr _ _ min | After 3 min standing | | | | |
| **ACTIVITY** Time: _ _ hr _ _min; Details of activity*: | | | | | |
| _ _ hr _ _ min | Before activity | | | | |
| _ _ hr _ _ min | After 3 min activity | | | | |
| **LUNCH** Time: _ _ hr _ _min; Details of food/fluid*: | | | | | |
| _ _ hr _ _ min | Lying | | | | |
| _ _ hr _ _ min | After 3 min standing | | | | |
| **ACTIVITY** Time: _ _ hr _ _min; Details of activity*: | | | | | |
| _ _ hr _ _ min | Before activity | | | | |
| _ _ hr _ _ min | After 3 min activity | | | | |
| **DINNER** Time: _ _ hr _ _min; Details of food/fluid *: | | | | | |
| _ _ hr _ _ min | Lying | | | | |
| _ _ hr _ _ min | After 3 min standing | | | | |

For peer review only



For peer review only - http://bmjopen.bmj.com/site/about/guidelines.xhtml

| **BEFORE SLEEPING (IN BED)** Time: _ _ hr _ _min | | | | | |
|---|---|---|---|---|---|
| 22.15pm<br>**(In bed)** | Lying in usual sleeping position (as with pillows) | | | | |

**Measure sitting BP/HR only if you find it difficult to stand.**

**Please record any other type of activity that you would like to tell us about and is not listed above, with time & position.**

| Enter time | Position/Activity | Blood Pressure | Heart Rate | Symptoms | Other details |
|---|---|---|---|---|---|
| _ _ hr _ _ min | | | | | |
| _ _ hr _ _ min | | | | | |

For peer review only



For peer review only - http://bmjopen.bmj.com/site/about/guidelines.xhtml

# Adapted Autonomic Profile (aAP) protocol

### What does it entail?

Measuring blood pressure (BP) and heart rate (HR) at key times as outlined below while at home, with a personal approved home BP/HR monitor. An example is Omron, approved by the British Hypertension Society. The recordings provide information on how your autonomic nervous system responds to key activities in daily life such as postural change, before/after food and exertion. Experience over the decades indicates that it provides adequate data for initial diagnosis and for guidance on treatment.

Please record time, position, BP and HR, and key symptoms (such as dizziness) in brief on the accompanying **aAP diary sheet**. This is of particular importance in autonomic conditions and differs substantially from BP/HR recordings commonly used for high BP. Recordings should be taken on waking, after meals, after exertion and before sleep as outlined below:

- **WAKING** - Take a measurement lying, then after 3 minutes of sitting, then after 3 minutes of standing.

- **EATING** - After a standard meal (breakfast, lunch or dinner), within 10-15 minutes, take a measurement lying, then after 3 minutes of standing. Please note down what food and drink you have consumed (including alcohol) in the space provided.

- **EXERTION** - take a measurement After 3-5 mins of activity (physical, cognitive or emotional) morning and afternoon, separated from lunch and dinner. **NB:** exercise exertion levels will be different for everyone, an example of **physical exertion** can be 5 minutes of gentle walking, or up and down a flight of stairs. An example of **emotional exertion** might be watching an exciting sporting match or film. **Cognitive exertion** might be 5 minutes working out a crossword puzzle. We prefer everyone to attempt at least one form of physical exertion if possible. Please discuss with the clinical team what form of exercise or exertion may be most appropriate for you.

Note that the only reading which we would like you to do seated is the waking reading – a measurement initially on lying, then at sitting for 3 minutes and then at standing for 3 minutes. If it is difficult to stand at other times substitute sitting for standing, especially if after exertion or food.

If you wish to add additional activities, which worsens your symptoms, record them with the time, event/activity and position (lying, sitting, standing).

### Does it involve preparation?

Ensure that you choose a day when you can complete all of the measurements on the record sheet. It is intended to provide relevant autonomic information during a standard day with usual activities, and thus no change in schedule is needed. The aAP can be repeated on another day if needed.



For peer review only - http://bmjopen.bmj.com/site/about/guidelines.xhtml

**What are the advantages of doing the test ?**

The test will inform the clinician about the response of your autonomic system to some of the common triggers or stimuli in daily life. The test also helps you understand what makes your autonomic symptoms worse, which might help you modify some of these activities or triggers.

**Are there any risks of doing the test?**

There is a chance that standing may cause dizziness or even fainting for some people, so please ensure you are leaning against a wall when checking BP/HR on standing. If possible, have another person present in the room whilst performing the test standing. Abandon the test and sit or lie down if symptoms are worse.

**Does it cause discomfort, and are there after-effects?**

The BP cuff may feel uncomfortably tight for a short period if you have a high BP, as some may do, especially while lying down. There should be no after-effects.

**Where does it take place?**

The test can be undertaken in your own home and independent of the GP surgery or hospital. This avoids travel and can be performed whenever convenient. And it can be repeated to determine the effects of treatment.

**How is the result/event sheet forwarded?**

Please enter the results along with your name/ number and the date in the diary sheet and email or post to the clinician/ service:

Address

***The BP/HR autonomic profile and protocol originally was devised and evaluated for autonomic conditions by Professor Mathias, when he directed and developed the UK National Autonomic Referral Units, at St Mary's Hospital & the National Hospital for Neurology & Neurosurgery @ Queen Square in London. It has been adapted for home use in this protocol and has been of value in the Coronavirus era and its aftermath.***

**June 2022**



For peer review only - http://bmjopen.bmj.com/site/about/guidelines.xhtml

# Modified COVID-19 Yorkshire Rehabilitation Screening (C19-YRS)

## Self-report version

Participant Identification Number:

HEARTLOC C19YRS form number:

Date: Time:

*The purpose of this questionnaire is to find out more about your current problems following COVID-19 illness. Your responses will be recorded in your clinical notes. We will use this information to monitor your symptoms, offer treatments and assess response to treatment.*

*This questionnaire will take around 15 minutes. If there are any topics you don't want to talk about you can choose not to respond.*

*Do you consent for this information to be used for audit and research as well ?* **Yes** ☐ **No** ☐

**SYMPTOM SEVERITY**

*Please answer the questions below to the best of your knowledge.*
*'Now' refers to how you feel now/this week (last 7 days).*
*"Pre-COVID" refers to how you were feeling prior to contracting the illness.*
*If you are unable to recall this, just state 'don't know'*

*Rate the severity of each problem on a scale of 0-3:*
***0 = None; no problem***
***1 = Mild problem; does not affect daily life***
***2 = Moderate problem; affects daily life to a certain extent***
***3 = Severe problem; affects all aspects of daily life; life-disturbing***

| | | | |
|---|---|---|---|
| 1. Breathlessness | Breathlessness: | **Now** | **Pre-COVID** |
| | a) At rest | **0** ☐ **1** ☐ **2** ☐ **3** ☐ | **0** ☐ **1** ☐ **2** ☐ **3** ☐ |
| | b) Changing position e.g. from lying to sitting or sitting to lying | **0** ☐ **1** ☐ **2** ☐ **3** ☐ | **0** ☐ **1** ☐ **2** ☐ **3** ☐ |
| | c) On dressing yourself | **0** ☐ **1** ☐ **2** ☐ **3** ☐ | **0** ☐ **1** ☐ **2** ☐ **3** ☐ |
| | d) On walking up a flight of stairs | **0** ☐ **1** ☐ **2** ☐ **3** ☐ | **0** ☐ **1** ☐ **2** ☐ **3** ☐ |
| 2. Cough/ throat sensitivity/ voice change | Cough/ throat sensitivity | **0** ☐ **1** ☐ **2** ☐ **3** ☐ | **0** ☐ **1** ☐ **2** ☐ **3** ☐ |
| | Change of voice | **0** ☐ **1** ☐ **2** ☐ **3** ☐ | **0** ☐ **1** ☐ **2** ☐ **3** ☐ |
| 3. Fatigue (tiredness | Fatigue levels in your usual activities | **0** ☐ **1** ☐ **2** ☐ **3** ☐ | **0** ☐ **1** ☐ **2** ☐ **3** ☐ |



For peer review only - http://bmjopen.bmj.com/site/about/guidelines.xhtml

| | | | |
|---|---|---|---|
| not improved by rest) | | | |
| 4. Smell/taste | Altered smell | **0** ☐ **1** ☐ **2** ☐ **3** ☐ | **0** ☐ **1** ☐ **2** ☐ **3** ☐ |
| | Altered taste | **0** ☐ **1** ☐ **2** ☐ **3** ☐ | **0** ☐ **1** ☐ **2** ☐ **3** ☐ |
| 5. Pain/discomfort | Chest pain | **0** ☐ **1** ☐ **2** ☐ **3** ☐ | **0** ☐ **1** ☐ **2** ☐ **3** ☐ |
| | Joint pain | **0** ☐ **1** ☐ **2** ☐ **3** ☐ | **0** ☐ **1** ☐ **2** ☐ **3** ☐ |
| | Muscle pain | **0** ☐ **1** ☐ **2** ☐ **3** ☐ | **0** ☐ **1** ☐ **2** ☐ **3** ☐ |
| | Headache | **0** ☐ **1** ☐ **2** ☐ **3** ☐ | **0** ☐ **1** ☐ **2** ☐ **3** ☐ |
| | Abdominal pain | **0** ☐ **1** ☐ **2** ☐ **3** ☐ | **0** ☐ **1** ☐ **2** ☐ **3** ☐ |
| 6. Cognition | Problems with concentration | **0** ☐ **1** ☐ **2** ☐ **3** ☐ | **0** ☐ **1** ☐ **2** ☐ **3** ☐ |
| | Problems with memory | **0** ☐ **1** ☐ **2** ☐ **3** ☐ | **0** ☐ **1** ☐ **2** ☐ **3** ☐ |
| | Problems with planning | **0** ☐ **1** ☐ **2** ☐ **3** ☐ | **0** ☐ **1** ☐ **2** ☐ **3** ☐ |
| 7. Palpitations/ dizziness | Palpitations in certain positions, activity or at rest | **0** ☐ **1** ☐ **2** ☐ **3** ☐ | **0** ☐ **1** ☐ **2** ☐ **3** ☐ |
| | Dizziness in certain positions, activity or at rest | **0** ☐ **1** ☐ **2** ☐ **3** ☐ | **0** ☐ **1** ☐ **2** ☐ **3** ☐ |
| 8. Post-exertional malaise (worsening of symptoms) | Crashing or relapse hours or days after physical, cognitive or emotional exertion | **0** ☐ **1** ☐ **2** ☐ **3** ☐ | **0** ☐ **1** ☐ **2** ☐ **3** ☐ |
| 9. Anxiety/ mood | Feeling anxious | **0** ☐ **1** ☐ **2** ☐ **3** ☐ | **0** ☐ **1** ☐ **2** ☐ **3** ☐ |
| | Feeling depressed | **0** ☐ **1** ☐ **2** ☐ **3** ☐ | **0** ☐ **1** ☐ **2** ☐ **3** ☐ |
| | Having unwanted memories of your illness or time in hospital | **0** ☐ **1** ☐ **2** ☐ **3** ☐ | **0** ☐ **1** ☐ **2** ☐ **3** ☐ |
| | Having unpleasant dreams about your illness or time in hospital | **0** ☐ **1** ☐ **2** ☐ **3** ☐ | **0** ☐ **1** ☐ **2** ☐ **3** ☐ |
| | Trying to avoid thoughts or feelings about your illness or time in hospital | **0** ☐ **1** ☐ **2** ☐ **3** ☐ | **0** ☐ **1** ☐ **2** ☐ **3** ☐ |
| 10. Sleep | Sleep problems, such as difficulty falling asleep, staying asleep or oversleeping | **0** ☐ **1** ☐ **2** ☐ **3** ☐ | **0** ☐ **1** ☐ **2** ☐ **3** ☐ |



For peer review only

**FUNCTIONAL ABILITY**

| | | | |
|---|---|---|---|
| 11. Communication | Difficulty with communication/word finding difficulty/understanding others | **Now**<br>**0** ☐ **1** ☐ **2** ☐ **3** ☐ | **Pre-COVID**<br>**0** ☐ **1** ☐ **2** ☐ **3** ☐ |
| 12. Walking or moving around | Difficulties with walking or moving around | **0** ☐ **1** ☐ **2** ☐ **3** ☐ | **0** ☐ **1** ☐ **2** ☐ **3** ☐ |
| 13. Personal care | Difficulties with personal tasks such as using the toilet or getting washed and dressed | **0** ☐ **1** ☐ **2** ☐ **3** ☐ | **0** ☐ **1** ☐ **2** ☐ **3** ☐ |
| 14. Other activities of Daily Living | Difficulty doing wider activities, such as household work, leisure/sporting activities, paid/unpaid work, study or shopping | **0** ☐ **1** ☐ **2** ☐ **3** ☐ | **0** ☐ **1** ☐ **2** ☐ **3** ☐ |
| 15. Social role | Problems with socialising/interacting with friends* or caring for dependants<br><br>*related to your illness and not due to social distancing/lockdown measures | **0** ☐ **1** ☐ **2** ☐ **3** ☐ | **0** ☐ **1** ☐ **2** ☐ **3** ☐ |

**OTHER SYMPTOMS**

Please select any of the following symptoms you have experienced since your illness in the last 7 days.Please also select any previous problems that have worsened for you following your illness.

☐ Fever
☐ Skin rash/ discolouration of skin
☐ New allergy such as medication, food etc
☐ Hair loss
☐ Skin sensation (numbness/tingling/itching/nerve pain)
☐ Dry eyes/ redness of eyes
☐ Swelling of feet/ swelling of hands
☐ Easy bruising/ bleeding
☐ Visual changes
☐ Difficulty swallowing solids
☐ Difficulty swallowing liquids
☐ Balance problems or falls
☐ Weakness or movement problems or coordination problems in limbs
☐ Tinnitus
☐ Nausea
☐ Dry mouth/mouth ulcers
☐ Acid Reflux/heartburn
☐ Change in appetite
☐ Unintentional weight loss
☐ Unintentional weight gain
☐ Bladder frequency,urgency or incontinence
☐ Constipation, diarrhoea or bowel incontinence



For peer review only - http://bmjopen.bmj.com/site/about/guidelines.xhtml

For peer review only

☐ Change in menstrual cycles or flow
☐ Waking up at night gasping for air (also called sleep apnea)
☐ Thoughts about harming yourself

Other symptoms – free text

**OVERALL HEALTH**

How good or bad is your health overall in the last 7 days?

For this question, a score of 10 means the BEST health you can imagine. 0 means the WORST health you can imagine.

a) Now: WORST HEALTH 0 ☐ 1 ☐ 2 ☐ 3 ☐ 4 ☐ 5 ☐ 6 ☐ 7 ☐ 8 ☐ 9 ☐ 10 ☐ BEST HEALTH
b) Pre-Covid: WORST HEALTH 0 ☐ 1 ☐ 2 ☐ 3 ☐ 4 ☐ 5 ☐ 6 ☐ 7 ☐ 8 ☐ 9 ☐ 10 ☐ BEST HEALTH

**EMPLOYMENT**

Occupation: ________________________

Has your COVID-19 illness affected your work??

☐ No change
☐ On reduced working hours
☐ On sickness leave
☐ Changes made to role/ working arrangements (such as working from home or lighter duties)
☐ Had to retire/ change job
☐ Lost job

Any other comments/concerns:_______________________________________________

**PARTNER/FAMILY/CARER PERSPECTIVE**

This is space for your partner, family or carer to add anything from their perspective:



For peer review only - http://bmjopen.bmj.com/site/about/guidelines.xhtml

BMJ Open

# HEART rate variability biofeedback for LOng Covid symptoms (HEARTLOC): protocol for a feasibility study

| | |
|---|---|
| Journal: | *BMJ Open* |
| Manuscript ID | bmjopen-2022-066044.R1 |
| Article Type: | Protocol |
| Date Submitted by the Author: | 26-Oct-2022 |
| Complete List of Authors: | Corrado, Joanna; University of Leeds, Academic Department of Rehabilitation Medicine; Leeds Teaching Hospitals NHS Trust<br>Halpin, Stephen; University of Leeds<br>Preston, Nick; University of Leeds<br>Whiteside, Diana; Leeds Community Healthcare NHS Trust<br>Tarrant, Rachel; Leeds Community Healthcare NHS Trust<br>Davison, Jennifer; Leeds Community Healthcare NHS Trust<br>Simms, Alexander; Leeds Teaching Hospitals NHS Trust<br>O'Connor, Rory J.; University of Leeds, Academic Department of Rehabilitation Medicine; Leeds Teaching Hospitals NHS Trust, National Demonstration Centre in Rehabilitation<br>Casson, Alexander; The University of Manchester<br>Sivan, Manoj; University of Leeds, Academic Department of Rehabilitation Medicine; Leeds Teaching Hospitals NHS Trust |
| <b>Primary Subject Heading</b>: | Rehabilitation medicine |
| Secondary Subject Heading: | Infectious diseases |
| Keywords: | COVID-19, Health informatics < BIOTECHNOLOGY & BIOINFORMATICS, REHABILITATION MEDICINE, CARDIOLOGY |
| | |

SCHOLARONE™
Manuscripts

# HEART rate variability biofeedback for LOng Covid symptoms (HEARTLOC): protocol for a feasibility study

**Corrado J[1,2,3], Halpin SJ[1,2,3], Preston N[1], Whiteside D[2], Tarrant R[2], Davison J[2], Simms AD[2,4], O'Connor RJ[1,3], Casson AJ[1,4,5], Sivan M[1,2,3]**

*Affiliations:*

[1] Academic Department of Rehabilitation Medicine, Leeds Institute of Rheumatic and Musculoskeletal Medicine, University of Leeds

[2] Covid rehabilitation service, Leeds Community Healthcare NHS Trust

[3] National Demonstration Centre of Rehabilitation Medicine, Leeds Teaching Hospitals NHS Trust

[4] Department of Cardiology, Leeds Teaching Hospitals NHS Trust

[5] Department of Electrical and Electronic Engineering, University of Manchester

[4] Manchester the Henry Royce Institute, Manchester, United Kingdom

**Correspondence:**

Dr Manoj Sivan MD FRCP Ed

Academic Department of Rehabilitation Medicine

Leeds Institute of Rheumatic and Musculoskeletal Medicine

University of Leeds

Martin Wing, Leeds General Infirmary, LS1 3EX

Tel: 0044 1133922564

E-mail address: m.sivan@leeds.ac.uk

**Word count:** 2930 (excluding abstract and references)

For peer review only

# Abstract (277/300)

## Introduction

Long covid (LC), also known as Post-COVID-19 syndrome, refers to symptoms persisting 12 weeks after COVID-19 infection. It affects up to 1 in 7 people contracting the illness and causes a wide range of symptoms, including fatigue, breathlessness, palpitations, dizziness, pain and brain fog. Many of these symptoms can be linked to dysautonomia or dysregulation of the autonomic nervous system after SARS-CoV2 infection. This study aims to test the feasibility and estimate the efficacy, of the Heart Rate Variability Biofeedback (HRV-B) technique via a standardised slow diaphragmatic breathing programme in individuals with LC.

## Methods and Analysis

30 adult LC patients with symptoms of palpitations or dizziness and an abnormal NASA Lean Test (NLT) will be selected from a specialist Long COVID rehabilitation service. They will undergo a 4-week HRV-B intervention using a Polar chest strap device linked to the Elite HRV phone application while undertaking the breathing exercise technique for two 10-min periods every day for at least 5 days a week. Quantitative data will be gathered during the study period using: HRV data from the chest strap and wrist-worn Fitbit, the modified COVID-19 Yorkshire Rehabilitation Scale (C19-YRSm), composite autonomic symptom score (COMPASS 31), World Health Organisation Disability Assessment Schedule (WHODAS 2.0) and EQ-5D-5L health related quality of life measures. Qualitative feedback on user experience and feasibility of using the technology in a home setting will also be gathered. Standard statistical tests for correlation and significant difference will be used to analyse the quantitate data.

## Ethics and Dissemination

The study has received ethical approval from Health Research Authority (HRA) Leicester South Research Ethics Committee (21/EM/0271). Dissemination plans include academic and lay publications.


For peer review only

## Study Registration

Clinicaltrials.gov No: NCT05228665

## Keywords

Post-COVID-19 condition, post-COVID-19 syndrome, dysautonomia, autonomic dysfunction, sympathetic, parasympathetic, rehabilitation, technology

# Article summary

## Strengths and limitations of the study

- To our knowledge, this is the first study of HRVB in long covid and will provide new information regarding the feasibility of the technology-based intervention in this condition.
- The estimation of efficacy will determine the scope and sample size for a larger controlled trial in the condition that currently has no definitive treatments
- The study will provide preliminary evidence on the correlation between long covid symptoms and dysautonomia.
- The limitation of this study is the small sample size of 30 participants which might not give an accurate estimate of efficacy.
- HRV-B is a technology-based intervention, therefore its take-up could be limited in those with a lack of experience in using digital technology in daily life, particularly those from less privileged backgrounds.

# Introduction

Post-COVID-19 syndrome or Long covid (LC) refers to persistent symptoms 12 weeks after SARS-COV2 infection and can include symptoms of physical fatigue, cognitive fatigue or "brain fog", breathlessness, pain and psychological distress.[1-3] An estimated 1.4 million people are reported to be affected by LC in the UK alone.[4] The condition can be highly debilitating for some, particularly middle-aged individuals who were previously functioning at

For peer review only

a high level and in demanding vocational roles.[5] Many will experience significant disruption to employment, social and caregiving roles, and participation in society.

Many LC symptoms such as palpitations, dizziness, fatigue, pain and breathlessness can be explained by the theory of dysautonomia.[6 7] This is a state of episodic dysregulation in the autonomic nervous system (ANS) with sympathetic overdrive and reduced parasympathetic activity. Dysautonomia plays a significant role in the symptomology of many long-term conditions including multiple sclerosis, Parkinson's disease, diabetes mellitus, fibromyalgia, chronic fatigue syndrome and migraine.[8]

One way of estimating and measuring autonomic function is through Heart Rate Variability (HRV), as cardiac rate and rhythm are controlled largely by the autonomic nervous system. The parasympathetic nervous system chiefly activates a slowing of heart rate through the vagus nerve, and the sympathetic response acts through the activation of β-adrenergic receptors.[9] HRV can be measured either in the time domain or frequency domain. HRV represents a measure of the variation in time between heartbeats (captured on an ECG strip as a time interval between the R waves of the QRS complexes). A low HRV is associated with sympathetic nervous system activation, also described as a state of 'fight or flight'. Higher HRV correspond with parasympathetic nervous system activation and is believed to reflect a state of rest and recovery. Lower HRV has been observed to be associated with fatigue and pain symptoms of chronic fatigue syndrome/myalgic encephalomyelitis (ME/CFS) and fibromyalgia [10-12], as well as other chronic physical and mental health pathologies including asthma, anxiety and stress.[10-14]

## Heart rate variability biofeedback (HRV-B)

When physiological parameters such as HRV are monitored in real-time with self-regulation techniques such as breathing exercises applied to influence the parameters, this is known as biofeedback (BFB).[15 16] In this study, for monitoring and modulating the HRV, we are utilising breathing techniques to encourage the predominance of parasympathetic nervous activity through vagus nerve activation. To the best of our knowledge, there have not yet been any studies of HRV-B in LC. However, HRV-B using breathing techniques has been tested in other

For peer review only

clinical conditions such as asthma[13], depression[17] and fibromyalgia[12]. A normal respiratory rate is between 12 and 20 breaths per minute.[18] The optimal breathing frequency to produce maximal increase in HRV varies for each individual but on average is between 5.5 and 6 breaths per minute and is known as resonant breathing.[13 18 19] Resonant breathing helps to restore autonomic balance due to baroreflex gain and vagal activation. [13 18-20]

There are several means of assessing HRV but most commonly these include the use of either wearable devices such as smartwatches or chest straps, or through small attachable Holter ECG units. These are non-invasive and readily available, although reliability differs between devices and platforms. Many commercial HRV devices are associated with smartphone app technology which can be readily downloaded and made available to participants for monitoring. Of the consumer grade devices available to monitor HRV the Polar H10 chest strap is felt to be the most reliable and remains accurate even during high-intensity activity.[21] The Polar H10 can be linked with the Elite HRV app which provides real time feedback on HRV and the user's response to breathing techniques. The combination of Polar H10 chest strap and Elite HRV app has been effectively used to harness real time physiological data, for example in athletes.[22] In contrast, many wrist worn devices such as Fitbit return a measure of HRV only while the user is asleep due to motion and other interference sources, meaning real-time HRV-B is not possible.

The aim of this study is to determine the feasibility and impact of a structured HRV-B regime incorporating diaphragmatic breathing exercise, on LC symptoms. We wish to test the acceptability and compliance of the intervention and estimate effect on symptoms using standardised validated measures of LC and dysautonomia.

## Aims and objectives

The aim of this study is: To assess the feasibility of a 4-week HRV biofeedback structured breathing programme in individuals with LC.

The objectives include:

1. Does breathing exercises through HRV-B increase HRV amongst participants with LC?

For peer review only

2. Are consumer grade monitors appropriate technology to use for HRV-B in the domiciliary setting?
3. Does regular HRV-B have any effect on LC symptoms?

# Methods

## Study design

This is a phase 2 uncontrolled open-label feasibility study of a home technology-based HRV-B in 30 individuals with LC. Potential participants will be identified through the Leeds COVID-19 Rehabilitation Service, based at Leeds Community Healthcare NHS Trust. The study period will be 6 weeks for each participant. The study start date is 24th Jan 2022, and the anticipated end date is 31st March 2024.

## Eligibility criteria

The inclusion criteria are

- Age > or = 18 years
- Confirmed LC diagnosis as per the NICE criteria for post-COVID syndrome [1]
- Self-rating of at least ‘moderate’ or ’severe’ on dysautonomia questions of palpitations or dizziness on the C19-YRSm [23]; and
- Abnormal NASA Lean Test (NLT)[24-26]
    - HR increase of 30bpm or ≥120bpm
      or
    - BP decrease of 20mmHg systolic or 10 mmHg diastolic in the first 3 minutes of standing

NLT is an accepted measure of cardiovascular instability and is conducted at initial assessment clinic for all LC service users in the Leeds COVID rehabilitation service. The patient lies down for 2 to five minutes prior to the test with HR and BP taken each minute to calculate average supine values. They then stand with heels 6 inches from a wall and lean back against it with HR and BP taken each minute for 10 min. Abnormal results (as described above) are demonstrated through orthostatic hypotension or tachycardia on standing which are hallmarks of dysautonomia and therefore objectively quantifiable. The participants who have dysautonomia symptoms but do not meet the mentioned thresholds will not be

For peer review only

included in this feasibility study but will be potential recruits for future larger scale studies using the same intervention.

The exclusion criteria are

- Unable to use the wearable or smartphone app technology
- Cognitive difficulties or mental health disorders causing inability to consent
- Any cardiac arrhythmias that are being planned for further investigations and specialist management in the Cardiology service
- Any unstable cardiorespiratory disease which needs further medical interventions (except asthma management)

## Equipment and Technology

To collect medium-term HRV data, participants will wear a Fitbit Charge 5 smartwatch for a total of 6 weeks. The HRV-B itself will be conducted using a Polar H10 chest strap for 10 minutes twice daily. This connects via Bluetooth to the Elite HRV smartphone app which is downloaded to participants' phones. Participants will aim to increase their HRV score as displayed in Elite HRV in real time using a diaphragmatic breathing technique (Figure 1). Omron M2 blood pressure monitor (endorsed by the British Hypertension Society) will be used to conduct NASA Lean test (NLT) in clinic and the adapted Autonomic Profile (aAP)[27].

Insert Fig 1 about here

## Study phases

The study will be carried out in the following three phases:

- Pre HRV-B phase
- HRV-B phase
- Post HRV-B phase

### Pre HRV-B phase

The participant will either be invited to a research clinic or visited at their home by a member of the research team (first appointment A1). They would have already received the participant information sheet (PIS) at screening and would have had more than 24 hours to read and understand the content of the PIS. Written consent will be signed by the participant

For peer review only

and the researcher during this first visit. Devices and baseline outcome measures used in this stage are:

- Fitbit charge 5 device and the Fitbit smartphone application: The participant will be requested to have the Fitbit device on most of the time during the 6-week period. The application records HRV at night along with other measures of sleep (sleep stages, HR) and daytime activity (such as step count).
- C19-YRSm: The COVID-19 Yorkshire Rehabilitation Scale (C19YRS) is the literature's first condition-specific patient recorded outcome measure which has been validated in the LC population.[28 29] The modified scale provides a symptom severity score (out of 30), functional disability score (out of 15), other symptoms score (out of 25) and overall health score (out of 10).[23] The participant will complete C19YRSm (Supplementary file 1) at weekly intervals to monitor the impact of the intervention on LC symptoms. They will also have weekly telephone reviews with study researchers for troubleshooting and to ensure maximal compliance with the study.
- COMPASS (Composite Autonomic Symptom Score): The COMPASS 31 will be completed by the participant at the initial visit and again 6 weeks later at the end of the study. Autonomic symptoms are scored for different domains including orthostatic intolerance, vasomotor, secretomotor, gastrointestinal, bladder and pupillomotor. Total scores for each domain are multiplied by a set weighting and then added together to provide a score out of 100 representing severity of autonomic symptoms. A higher score represents greater severity.[30]
- Adapted Autonomic Profile (aAP): This is an autonomic profile test developed by St Mary's Hospital and the National Hospital for Neurology and Neurosurgery and later adapted for domiciliary use during the COVID-19 pandemic.[27] Participants are asked to monitor their heart rate and blood pressure on lying, and at 3 minutes of standing at various intervals over 24 hours, including after waking, after eating breakfast/ lunch/ dinner, before and after 5 minutes of exercise, and before bed (Supplementary file 2). Abnormal results are calculated using the same criteria for heart rate and blood pressure differences as the NLT (HR increase > 30/min or BP drop >20 mm Hg).
- World Health Organisation Disability Assessment Schedule (WHODAS): This is validated generic measure of functioning and disability. The 36-item scale captures six

For peer review only

domains of life (cognition, mobility, self-care, getting along, life activities and participation) with a summary score ranging from 0 (no disability) to 100 (full disability)[31 32]

- EQ-5D-5L: The EQ-5D-5L instrument, provided by the EuroQol Group, is one of widely used quality of life measures, consists of five items covering: mobility, self-care, usual activities, pain/discomfort, and anxiety/depression.[33] The item scores can be converted into a total index score by applying health preference weights elicited from a general population. This index score can also be used in economic evaluations to assess the cost-effectiveness of health interventions.[34]

The A1 appointment will last approximately 2 hours and may be longer for those with cognitive fatigue or 'brain fog'. If felt necessary, it will be divided into two one-hour visits to reduce cognitive fatigue.

### HRV-B phase

One week after the A1 appointment, the participant will be either invited to attend a research clinic or visited at home by a researcher (second appointment A2) to commence the HRV-B study phase. This involves:

- Polar H10 chest strap and Elite HRVB application: The participant will be familiarised with the technology and introduced to a paced breathing regimen via a one-to-one demonstration. They will be instructed to perform the breathing technique using the application at least twice a day, 10 minutes per session, for a period of 4 weeks. The chest strap device will record HRV for the duration of the session, and the data gets recorded in the application. Whilst this phase is ongoing, participants will continue to wear the Fitbit Charge 5 device for the duration of this phase.
- Fitbit charge 5 device and the Fitbit smartphone application
- C19-YRSm

### Post HRV-B phase

The participant will be asked to stop the HRV-B intervention after completing 4 weeks of the treatment. They will be asked to continue using the Fitbit device for another week when not doing the intervention. They will then either be invited to a research clinic or be visited at home by a study researcher. At this appointment (A3), the participant will complete:

For peer review only - http://bmjopen.bmj.com/site/about/guidelines.xhtml

- C19 YRSm The C19-YRSm will be completed by the patient every week for a total of 6 weeks. There will be a total of 7 C19-YRS documents completed.
- Fitbit charge 5 device and the Fitbit smartphone application
- NLT and aAP
- COMPASS 31
- WHODAS
- EQ-5D-5L

During the A3 appointment, the Polar H10 strap and the Fitbit device will be retrieved. The participants will be invited to complete a further C19-YRS, by email or postal four weeks after completion aAP for 24 hours and to email or post the results to the study researcher.

## Outcome measures

**The primary outcome measure** is the C19YRSm, a self-reported patient-reported outcome measure to assess LC symptom severity, functional disability, and overall health status.

**Secondary outcome measures** include:

***Heart rate measures from chest strap:***

- 7-day average HRV score out of 100 - quantified by the Elite HRV app via the root mean square of successive differences between normal heartbeats (rMSSD). A natural log (ln) is applied to this figure and then expanded to generate a 1 to 100 score
- Mean R-R interval
- Heart rate
- rMSSD
- SDNN (standard deviation of NN intervals)
- Total Power
- Low frequency power (LF)
- High frequency power (HF)
- LF:HF ratio

***Fitbit Data:***

- Sleep staging data
- Resting heart rate
- Daily activity levels e.g. step count and exercise type and duration

For peer review only

***Patient Reported Outcome Measures:***

- NLT and aAP
- COMPASS 31
- WHODAS
- EQ-5D-5L

During our final interaction with participants in the study, we will ask them the following questions to assess the feasibility of the study:

1. "How did you find using the technology?"
2. "How did you find the breathing intervention?"
3. "Have you noticed any change in your symptoms?"

Their opinions and suggestions will be recorded as quotes in their participant files. However, we are not planning to undertake a formal qualitative analysis of responses as it is not one of the main objectives of this study.

A summary of the schedule for the completion of outcome measures is shown in Table 1.

Insert Table 1 here

Table 1. Outcome measures summary schedule

| | Initial assessment Clinic | Pre HRV-B phase (1 week) | HRV-B phase (4 weeks) | Post HRV-B phase (1 week) |
|---|---|---|---|---|
| Autonomic screening (NLT) | √ | | | √ |
| Autonomic function (COMPASS 31) | | √ | | √ |
| Home autonomic test (aAP) | √ | | | √ |
| Fitbit wrist strap HRV, sleep data | | √ | √ daily | √ |
| Polar H10 chest strap HRV data | | | √ daily | |
| LC specific PROM C19-YRSm | | √ | √ weekly | √ |
| Daily function (WHODAS) | | √ | | √ |

For peer review only

| | | | | |
|---|---|---|---|---|
| Quality of life (EQ5D-5L) | | √ | | √ |

## Sample size

A formal sample size calculation is not required for a feasibility study as it does not mimic a definitive randomised trial and aim is not to measure effect size.[35] A sample size of 30 is the average sample size across feasibility studies and is accepted as reasonable size to assess the acceptability and suitability of the intervention.[36]

## Statistical Analysis

Quantitative data from standardised questionnaires will be scored as per standard procedures. Data downloaded from the wearable devices will be extracted, cleaned, and summarised using specific software packages, including Matlab and Python. Quantitative data will be analysed with simple descriptive statistics. The presence and magnitude of pre and post-intervention differences will be examined using repeated paired-sample T-tests (with Bonferroni adjustment for multiple comparisons), and the effect size will be explored using both ANOVA partial Eta squared, and Cohen's d. Additional exploratory analyses may also be performed to fully analyse the dataset produced, guided by the findings of the descriptive statistics.

## Patient and public involvement

Members of the patient advisory group with lived experience of long covid have been involved in the design, development, and delivery of the project. Members of the patient advisory group attended proposal research planning meetings and shared their experiences on symptoms of dysautonomia which helped shaped the research question, design and outcome measures of this study. Members of this group have contacts with wider patient community groups and helped disseminate information about the study. The advisory group meets quarterly with the research team to review progress, ensure the research continues to answer relevant issues and that findings can inform long covid care. The group will be

For peer review only

involved in the dissemination of research findings and writing lay summary reports that will be shared with the participants.

## Ethics and dissemination

The study has received ethical approval from Health Research Authority (HRA) Leicester South Research Ethics Committee (21/EM/0271). Informed consent will be obtained from all participants. Potential participants will have a minimum of 24 hours to review the PIS and discuss queries with the researcher prior to signing the written consent. GDPR rules will be strictly followed for all data gathered during the study. All data will be fully anonymised as soon as practical. All devices used are CE marked and are being used for their intended purposes. There is potential for minor skin irritation from wearing the Fitbit and Polar H10 devices. This will be enquired about at each weekly telephone review.

For participants with cognitive fatigue or 'brain fog' relating to LC, the length of the appointments with the researcher (A1, A2, A3) may be longer than normal. Supplementary written information will be provided, and if necessary, each of these appointments may be conducted in two shorter sessions to reduce information overload and possible impact on LC symptoms. Participants will be advised that they do not need to proceed with the appointments or the study if they do not want to. All appointments other than the initial NLT can occur at the participants' homes to reduce travel and inconvenience. Participants are free to withdraw at any point in the study. They will be encouraged to give reasons for the withdrawal, but it will not be compulsory to give a reason for withdrawal.

Dissemination will include both academic publications and lay summaries in various formats. Academic outputs will include both medical and engineering literature. Policy impact will be aided by our strong existing links to NHS England and the UK Long COVID National Task Force. Dr Sivan, who leads the NHR project Long Covid Multidisciplinary consortium for Optimising Treatments and Services across the NHS (LOCOMOTION)[37], is also advisor for the World Health Organisation (WHO - Europe) on COVID-19 rehabilitation and is also involved in the WHO working party developing a core set of outcome measures for LC.

For peer review only

## Conflicts of interest

Manoj Sivan is an advisor to the World Health Organisation (WHO) for the Long COVID policy in Europe.


## Acknowledgements

The authors would like to thank individuals with autonomic problems in long covid and healthcare professionals from the Leeds Covid Rehabilitation service who provided valuable suggestions and feedback during the iterative process of development of this protocol. We are grateful to the Patient Advisory Group for its involvement in all stages of this study.

## Funding statement

This research is supported by IAA EPSRC [Ref 112538] with University of Leeds as the sponsor organisation and the Leeds Community Healthcare NHS Trust Covid Rehabilitation service as the research site organisation.


## Data statement

We will use Open Science Framework (OSF) to share of all research outputs, including data, codes, and other types of information that has the potential to aid the advancement of scientific progress and benefit other researchers by adding transparency to the research process. Data will also be shared via the University of Leeds's public data repository to increase exposure. The OSF will consist of two levels: a data dictionary with basic info about the study, and a more detailed dataset (e.g., for further analysis/meta-analysis). Data will be issued with a Digital Object Identifier (DOI) which will allow it to be referenced and make it easier for others to identify and access relevant files.

For peer review only

## Supplementary files

1 Modified Covid Yorkshire Rehabilitation Scale (C19-YRSm) questionnaire

2 adapted Autonomic Profile (aAP) diary and instruction sheet

## Author contributions

Manoj Sivan and Alex Casson conceptualised the study. Manoj Sivan, Alex Casson and Rory J O'Connor were awarded EPSRC IAA pump-priming grant for the feasibility study with Manoj Sivan as the Principal Investigator. Joanna Corrado, Stephen Halpin, Nick Preston, Diana Whiteside, Rachel Tarrant, Jenny Davidson, Alexander J Simms, Rory J O'Connor, Alex J Casson and Manoj Sivan contributed to the study design and obtained ethical approval. Joanna Corrado and Manoj Sivan wrote an initial draft of the paper by adapting the grant proposal and the ethics protocol. All authors approved the final manuscript. All authors will contribute to recruitment, data acquisition and analysis of the study findings. Manoj Sivan is the corresponding author and guarantor.

## Figure legends

Fig 1. Heart Rate Variability Biofeedback (HRV-B) using a breathing technique and chest strap for real time HRV monitoring. Polar H10 picture from Wikimedia commons, reprinted under CC BY-SA 3.0 license. EliteHRV screenshot from Wikimedia commons, reprinted under CC BY-SA 4.0 license.

For peer review only

# References


1. National Institute for Health and Care Excellence (NICE) Scottish Intercollegiate Guidelines Network (SIGN) and Royal College of General Practitioners (RCGP). COVID-19 rapid guideline: managing the long- term effects of COVID-19. London: NICE 2022.
2. Crook H, Raza S, Nowell J, et al. Long covid—mechanisms, risk factors, and management. *bmj* 2021;374
3. World Health Organisation. Support for rehabilitation: self-management after COVID-19-related illness. Copenhagen: WHO Regional Office for Europe 2021.
4. Office of National Statistics. Prevalence of ongoing symptoms following coronavirus (COVID-19) infection in the UK: 1 June 2022. London: ONS 2022.
5. Statistics OoN. Coronavirus and the social impacts of 'long COVID' on people's lives in Great Britain: 7 April to 13 June 2021. 2021; https://www.ons.gov.uk/peoplepopulationandcommunity/healthandsocialcare/conditionsanddiseases/articles/coronavirusandthesocialimpactsoflongcovidonpeopleslivesingreatbritain/7aprilto13june2021. (accessed 02.05.2022).

6. Dani M, Dirksen A, Taraborrelli P, et al. Autonomic dysfunction in 'long COVID': rationale, physiology and management strategies. *Clin Med (Lond)* 2021;21(1):e63-e67. doi: 10.7861/clinmed.2020-0896 [published Online First: 2020/11/28]

7. Shouman K, Vanichkachorn G, Cheshire WP, et al. Autonomic dysfunction following COVID-19 infection: an early experience. *Clin Auton Res* 2021;31(3):385-94. doi: 10.1007/s10286-021-00803-8 [published Online First: 2021/04/17]

8. Zalewski P, Slomko J, Zawadka-Kunikowska M. Autonomic dysfunction and chronic disease. *Br Med Bull* 2018;128(1):61-74. doi: 10.1093/bmb/ldy036 [published Online First: 2018/11/13]

9. Heart rate variability. Standards of measurement, physiological interpretation, and clinical use. Task Force of the European Society of Cardiology and the North American Society of Pacing and Electrophysiology. *Eur Heart J* 1996;17(3):354-81. [published Online First: 1996/03/01]

10. Fournie C, Chouchou F, Dalleau G, et al. Heart rate variability biofeedback in chronic disease management: A systematic review. *Complement Ther Med* 2021;60:102750. doi: 10.1016/j.ctim.2021.102750 [published Online First: 2021/06/13]

11. Escorihuela RM, Capdevila L, Castro JR, et al. Reduced heart rate variability predicts fatigue severity in individuals with chronic fatigue syndrome/myalgic encephalomyelitis. *J Transl Med* 2020;18(1):4. doi: 10.1186/s12967-019-02184-z [published Online First: 2020/01/08]

12. Hassett AL, Radvanski DC, Vaschillo EG, et al. A pilot study of the efficacy of heart rate variability (HRV) biofeedback in patients with fibromyalgia. *Appl Psychophysiol Biofeedback* 2007;32(1):1-10. doi: 10.1007/s10484-006-9028-0 [published Online First: 2007/01/16]

13. Lehrer PM, Vaschillo E, Vaschillo B, et al. Biofeedback treatment for asthma. *Chest* 2004;126(2):352-61. doi: 10.1378/chest.126.2.352 [published Online First: 2004/08/11]

14. Goessl VC, Curtiss JE, Hofmann SG. The effect of heart rate variability biofeedback training on stress and anxiety: a meta-analysis. *Psychol Med* 2017;47(15):2578-86. doi: 10.1017/S0033291717001003 [published Online First: 2017/05/10]

For peer review only

15. Lehrer P, Vaschillo B, Zucker T, et al. Protocol for heart rate variability biofeedback training. *Biofeedback* 2013;41(3):98-109. doi: https://doi.org/10.5298/1081-5937-41.3.08

16. Gevirtz R. The promise of heart rate variability biofeedback: Evidence-based application. *Biofeedback* 2013;41(3):110-20. doi: https://doi.org/10.5298/1081-5937-41.3.01

17. Karavidas MK, Lehrer PM, Vaschillo E, et al. Preliminary results of an open label study of heart rate variability biofeedback for the treatment of major depression. *Appl Psychophysiol Biofeedback* 2007;32(1):19-30. doi: 10.1007/s10484-006-9029-z [published Online First: 2007/03/03]

18. Vaschillo EG, Vaschillo B, Lehrer PM. Characteristics of resonance in heart rate variability stimulated by biofeedback. *Appl Psychophysiol Biofeedback* 2006;31(2):129-42. doi: 10.1007/s10484-006-9009-3 [published Online First: 2006/07/14]

19. Lehrer PM, Gevirtz R. Heart rate variability biofeedback: how and why does it work? *Front Psychol* 2014;5:756. doi: 10.3389/fpsyg.2014.00756 [published Online First: 2014/08/08]

20. Pagaduan JC, Chen YS, Fell JW, et al. Can Heart Rate Variability Biofeedback Improve Athletic Performance? A Systematic Review. *J Hum Kinet* 2020;73:103-14. doi: 10.2478/hukin-2020-0004 [published Online First: 2020/08/11]

21. Gilgen-Ammann R, Schweizer T, Wyss T. RR interval signal quality of a heart rate monitor and an ECG Holter at rest and during exercise. *Eur J Appl Physiol* 2019;119(7):1525-32. doi: 10.1007/s00421-019-04142-5 [published Online First: 2019/04/21]

22. Flatt AA, Howells D. Effects of varying training load on heart rate variability and running performance among an Olympic rugby sevens team. *J Sci Med Sport* 2019;22(2):222-26. doi: 10.1016/j.jsams.2018.07.014 [published Online First: 2018/07/30]

23. Sivan M, Preston N, Parkin A, et al. The modified COVID-19 Yorkshire Rehabilitation Scale (C19-YRSm) patient-reported outcome measure for Long Covid or Post-COVID-19 syndrome. *J Med Virol* 2022 doi: 10.1002/jmv.27878 [published Online First: 2022/05/24]

24. Bungo MW, Charles JB, Johnson PC, Jr. Cardiovascular deconditioning during space flight and the use of saline as a countermeasure to orthostatic intolerance. *Aviat Space Environ Med* 1985;56(10):985-90. [published Online First: 1985/10/01]

For peer review only

25. Lee J, Vernon SD, Jeys P, et al. Hemodynamics during the 10-minute NASA Lean Test: evidence of circulatory decompensation in a subset of ME/CFS patients. *J Transl Med* 2020;18(1):314. doi: 10.1186/s12967-020-02481-y [published Online First: 2020/08/18]

26. Hyatt KH, Jacobson LB, Schneider VS. Comparison of 70 degrees tilt, LBNP, and passive standing as measrues of orthostatic tolerance. *Aviat Space Environ Med* 1975;46(6):801-8. [published Online First: 1975/06/01]

27. Sivan M, Corrado J, Mathias C. The adapted Autonomic Profile (aAP) Home-Based Test for the Evaluation of Neuro-Cardiovascular Autonomic Dysfunction. *Adv Clin Neurosci Rehabil* 2022 doi: https://doi.org/10.47795/QKBU6715

28. Sivan M, Halpin S, Gee J, et al. The self-report version and digital format of the COVID-19 Yorkshire Rehabilitation Scale (C19-YRS) for Long Covid or Post-COVID syndrome assessment and monitoring. *Advances in CLinical Neuroscience and Rehabilitation* 2021;20(3)

29. O'Connor RJ, Preston N, Parkin A, et al. The COVID-19 Yorkshire Rehabilitation Scale (C19-YRS): application and psychometric analysis in a post-COVID-19 syndrome cohort. *Journal of Medical Virology* 2022;94(3):1027-34.

30. Sletten DM, Suarez GA, Low PA, et al. COMPASS 31: a refined and abbreviated Composite Autonomic Symptom Score. *Mayo Clin Proc* 2012;87(12):1196-201. doi: 10.1016/j.mayocp.2012.10.013 [published Online First: 2012/12/12]

31. Garin O, Ayuso-Mateos JL, Almansa J, et al. Validation of the "World Health Organization Disability Assessment Schedule, WHODAS-2" in patients with chronic diseases. *Health Qual Life Outcomes* 2010;8:51. doi: 10.1186/1477-7525-8-51 [published Online First: 2010/05/21]

32. Ustun TB, Kostanjsek N, Chatterji S, et al. Measuring health and disability : manual for WHO Disability Assessment Schedule (WHODAS 2.0). 2010. https://www.who.int/publications/i/item/measuring-health-and-disability-manual-for-who-disability-assessment-schedule-(-whodas-2.0) (accessed 19.06.2022).

33. Herdman M, Gudex C, Lloyd A, et al. Development and preliminary testing of the new five-level version of EQ-5D (EQ-5D-5L). *Qual Life Res* 2011;20(10):1727-36. doi: 10.1007/s11136-011-9903-x [published Online First: 2011/04/12]

For peer review only

34. Brooks R. EuroQol: the current state of play. *Health Policy* 1996;37(1):53-72. doi: 10.1016/0168-8510(96)00822-6 [published Online First: 1996/06/06]

35. Whitehead AL, Sully BG, Campbell MJ. Pilot and feasibility studies: is there a difference from each other and from a randomised controlled trial? *Contemp Clin Trials* 2014;38(1):130-3. doi: 10.1016/j.cct.2014.04.001 [published Online First: 2014/04/17]

36. Billingham SA, Whitehead AL, Julious SA. An audit of sample sizes for pilot and feasibility trials being undertaken in the United Kingdom registered in the United Kingdom Clinical Research Network database. *BMC Med Res Methodol* 2013;13:104. doi: 10.1186/1471-2288-13-104 [published Online First: 2013/08/22]

37. Sivan M, Greenhalgh T, Darbyshire JL, et al. LOng COvid Multidisciplinary consortium Optimising Treatments and servIces acrOss the NHS (LOCOMOTION): protocol for a mixed-methods study in the UK. *BMJ Open* 2022;12(5):e063505. doi: 10.1136/bmjopen-2022-063505 [published Online First: 2022/05/18]

For peer review only

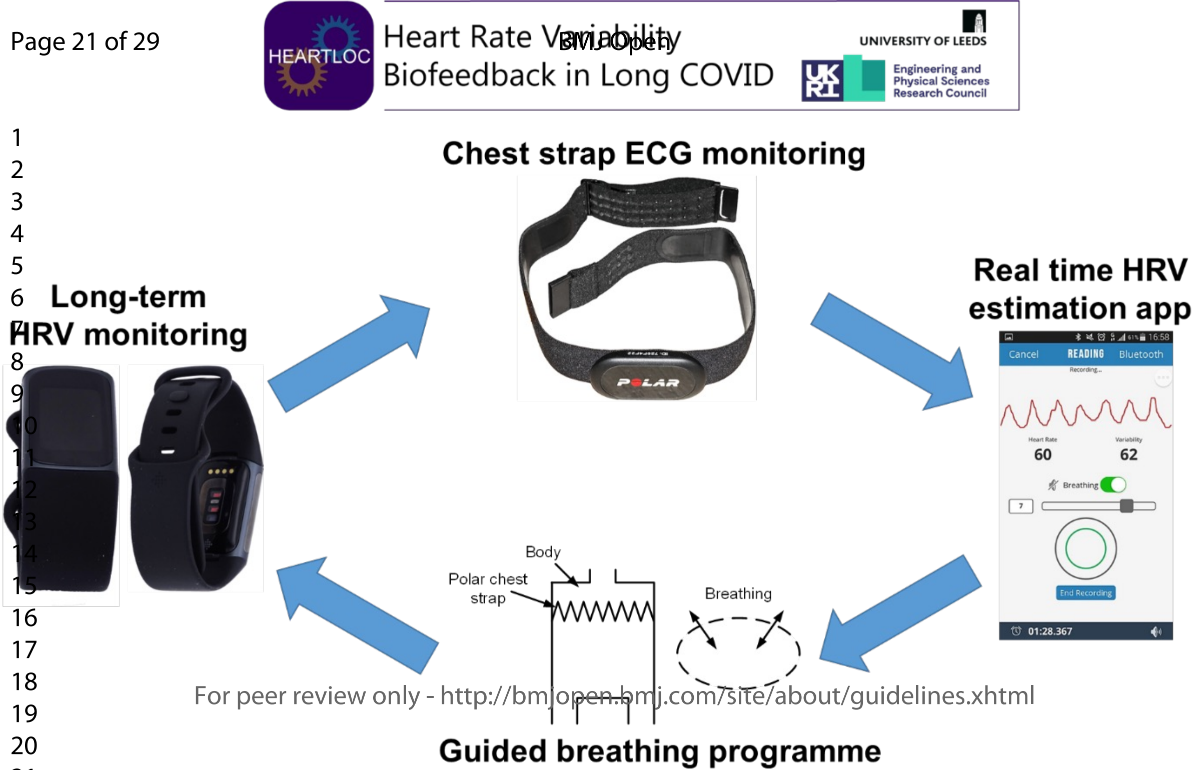


For peer review only - http://bmjopen.bmj.com/site/about/guidelines.xhtml

# Modified COVID-19 Yorkshire Rehabilitation Screening (C19-YRS)

## Self-report version

Participant Identification Number:

HEARTLOC C19YRS form number:

Date: Time:

*The purpose of this questionnaire is to find out more about your current problems following COVID-19 illness. Your responses will be recorded in your clinical notes. We will use this information to monitor your symptoms, offer treatments and assess response to treatment.*

*This questionnaire will take around 15 minutes. If there are any topics you don't want to talk about you can choose not to respond.*

*Do you consent for this information to be used for audit and research as well ?* **Yes** ☐ **No** ☐

**SYMPTOM SEVERITY**

*Please answer the questions below to the best of your knowledge.*
*'Now' refers to how you feel now/this week (last 7 days).*
*"Pre-COVID" refers to how you were feeling prior to contracting the illness.*
*If you are unable to recall this, just state 'don't know'*

*Rate the severity of each problem on a scale of 0-3:*
***0 = None; no problem***
***1 = Mild problem; does not affect daily life***
***2 = Moderate problem; affects daily life to a certain extent***
***3 = Severe problem; affects all aspects of daily life; life-disturbing***

| | | | |
|---|---|---|---|
| 1. Breathlessness | Breathlessness: | **Now** | **Pre-COVID** |
| | a) At rest | **0** ☐ **1** ☐ **2** ☐ **3** ☐ | **0** ☐ **1** ☐ **2** ☐ **3** ☐ |
| | b) Changing position e.g. from lying to sitting or sitting to lying | **0** ☐ **1** ☐ **2** ☐ **3** ☐ | **0** ☐ **1** ☐ **2** ☐ **3** ☐ |
| | c) On dressing yourself | **0** ☐ **1** ☐ **2** ☐ **3** ☐ | **0** ☐ **1** ☐ **2** ☐ **3** ☐ |
| | d) On walking up a flight of stairs | **0** ☐ **1** ☐ **2** ☐ **3** ☐ | **0** ☐ **1** ☐ **2** ☐ **3** ☐ |
| 2. Cough/ throat sensitivity/ voice change | Cough/ throat sensitivity | **0** ☐ **1** ☐ **2** ☐ **3** ☐ | **0** ☐ **1** ☐ **2** ☐ **3** ☐ |
| | Change of voice | **0** ☐ **1** ☐ **2** ☐ **3** ☐ | **0** ☐ **1** ☐ **2** ☐ **3** ☐ |
| 3. Fatigue (tiredness | Fatigue levels in your usual activities | **0** ☐ **1** ☐ **2** ☐ **3** ☐ | **0** ☐ **1** ☐ **2** ☐ **3** ☐ |



For peer review only - http://bmjopen.bmj.com/site/about/guidelines.xhtml

| | | | |
|---|---|---|---|
| not improved by rest) | | | |
| 4. Smell/taste | Altered smell | **0** ☐ **1** ☐ **2** ☐ **3** ☐ | **0** ☐ **1** ☐ **2** ☐ **3** ☐ |
| | Altered taste | **0** ☐ **1** ☐ **2** ☐ **3** ☐ | **0** ☐ **1** ☐ **2** ☐ **3** ☐ |
| 5. Pain/discomfort | Chest pain | **0** ☐ **1** ☐ **2** ☐ **3** ☐ | **0** ☐ **1** ☐ **2** ☐ **3** ☐ |
| | Joint pain | **0** ☐ **1** ☐ **2** ☐ **3** ☐ | **0** ☐ **1** ☐ **2** ☐ **3** ☐ |
| | Muscle pain | **0** ☐ **1** ☐ **2** ☐ **3** ☐ | **0** ☐ **1** ☐ **2** ☐ **3** ☐ |
| | Headache | **0** ☐ **1** ☐ **2** ☐ **3** ☐ | **0** ☐ **1** ☐ **2** ☐ **3** ☐ |
| | Abdominal pain | **0** ☐ **1** ☐ **2** ☐ **3** ☐ | **0** ☐ **1** ☐ **2** ☐ **3** ☐ |
| 6. Cognition | Problems with concentration | **0** ☐ **1** ☐ **2** ☐ **3** ☐ | **0** ☐ **1** ☐ **2** ☐ **3** ☐ |
| | Problems with memory | **0** ☐ **1** ☐ **2** ☐ **3** ☐ | **0** ☐ **1** ☐ **2** ☐ **3** ☐ |
| | Problems with planning | **0** ☐ **1** ☐ **2** ☐ **3** ☐ | **0** ☐ **1** ☐ **2** ☐ **3** ☐ |
| 7. Palpitations/ dizziness | Palpitations in certain positions, activity or at rest | **0** ☐ **1** ☐ **2** ☐ **3** ☐ | **0** ☐ **1** ☐ **2** ☐ **3** ☐ |
| | Dizziness in certain positions, activity or at rest | **0** ☐ **1** ☐ **2** ☐ **3** ☐ | **0** ☐ **1** ☐ **2** ☐ **3** ☐ |
| 8. Post-exertional malaise (worsening of symptoms) | Crashing or relapse hours or days after physical, cognitive or emotional exertion | **0** ☐ **1** ☐ **2** ☐ **3** ☐ | **0** ☐ **1** ☐ **2** ☐ **3** ☐ |
| 9. Anxiety/ mood | Feeling anxious | **0** ☐ **1** ☐ **2** ☐ **3** ☐ | **0** ☐ **1** ☐ **2** ☐ **3** ☐ |
| | Feeling depressed | **0** ☐ **1** ☐ **2** ☐ **3** ☐ | **0** ☐ **1** ☐ **2** ☐ **3** ☐ |
| | Having unwanted memories of your illness or time in hospital | **0** ☐ **1** ☐ **2** ☐ **3** ☐ | **0** ☐ **1** ☐ **2** ☐ **3** ☐ |
| | Having unpleasant dreams about your illness or time in hospital | **0** ☐ **1** ☐ **2** ☐ **3** ☐ | **0** ☐ **1** ☐ **2** ☐ **3** ☐ |
| | Trying to avoid thoughts or feelings about your illness or time in hospital | **0** ☐ **1** ☐ **2** ☐ **3** ☐ | **0** ☐ **1** ☐ **2** ☐ **3** ☐ |
| 10. Sleep | Sleep problems, such as difficulty falling asleep, staying asleep or oversleeping | **0** ☐ **1** ☐ **2** ☐ **3** ☐ | **0** ☐ **1** ☐ **2** ☐ **3** ☐ |



For peer review only

For peer review only - http://bmjopen.bmj.com/site/about/guidelines.xhtml

**FUNCTIONAL ABILITY**

| | | | |
|---|---|---|---|
| 11. Communication | Difficulty with communication/word finding difficulty/understanding others | **Now**<br>**0** ☐ **1** ☐ **2** ☐ **3** ☐ | **Pre-COVID**<br>**0** ☐ **1** ☐ **2** ☐ **3** ☐ |
| 12. Walking or moving around | Difficulties with walking or moving around | **0** ☐ **1** ☐ **2** ☐ **3** ☐ | **0** ☐ **1** ☐ **2** ☐ **3** ☐ |
| 13. Personal care | Difficulties with personal tasks such as using the toilet or getting washed and dressed | **0** ☐ **1** ☐ **2** ☐ **3** ☐ | **0** ☐ **1** ☐ **2** ☐ **3** ☐ |
| 14. Other activities of Daily Living | Difficulty doing wider activities, such as household work, leisure/sporting activities, paid/unpaid work, study or shopping | **0** ☐ **1** ☐ **2** ☐ **3** ☐ | **0** ☐ **1** ☐ **2** ☐ **3** ☐ |
| 15. Social role | Problems with socialising/interacting with friends* or caring for dependants<br><br>*related to your illness and not due to social distancing/lockdown measures | **0** ☐ **1** ☐ **2** ☐ **3** ☐ | **0** ☐ **1** ☐ **2** ☐ **3** ☐ |

**OTHER SYMPTOMS**

Please select any of the following symptoms you have experienced since your illness in the last 7 days.Please also select any previous problems that have worsened for you following your illness.

☐ Fever
☐ Skin rash/ discolouration of skin
☐ New allergy such as medication, food etc
☐ Hair loss
☐ Skin sensation (numbness/tingling/itching/nerve pain)
☐ Dry eyes/ redness of eyes
☐ Swelling of feet/ swelling of hands
☐ Easy bruising/ bleeding
☐ Visual changes
☐ Difficulty swallowing solids
☐ Difficulty swallowing liquids
☐ Balance problems or falls
☐ Weakness or movement problems or coordination problems in limbs
☐ Tinnitus
☐ Nausea
☐ Dry mouth/mouth ulcers
☐ Acid Reflux/heartburn
☐ Change in appetite
☐ Unintentional weight loss
☐ Unintentional weight gain
☐ Bladder frequency,urgency or incontinence
☐ Constipation, diarrhoea or bowel incontinence



For peer review only - http://bmjopen.bmj.com/site/about/guidelines.xhtml
For peer review only

☐ Change in menstrual cycles or flow
☐ Waking up at night gasping for air (also called sleep apnea)
☐ Thoughts about harming yourself

Other symptoms – free text

**OVERALL HEALTH**

How good or bad is your health overall in the last 7 days?

For this question, a score of 10 means the BEST health you can imagine. 0 means the WORST health you can imagine.

a) Now: WORST HEALTH 0 ☐ 1 ☐ 2 ☐ 3 ☐ 4 ☐ 5 ☐ 6 ☐ 7 ☐ 8 ☐ 9 ☐ 10 ☐ BEST HEALTH
b) Pre-Covid: WORST HEALTH 0 ☐ 1 ☐ 2 ☐ 3 ☐ 4 ☐ 5 ☐ 6 ☐ 7 ☐ 8 ☐ 9 ☐ 10 ☐ BEST HEALTH

**EMPLOYMENT**

Occupation: ________________________

Has your COVID-19 illness affected your work??

☐ No change
☐ On reduced working hours
☐ On sickness leave
☐ Changes made to role/ working arrangements (such as working from home or lighter duties)
☐ Had to retire/ change job
☐ Lost job

Any other comments/concerns:_________________________________________________

**PARTNER/FAMILY/CARER PERSPECTIVE**

This is space for your partner, family or carer to add anything from their perspective:



For peer review only - http://bmjopen.bmj.com/site/about/guidelines.xhtml

# The aAP diary sheet

## Participant Initials                                  Date

*__Food or fluid intake__ – please state what food or drink, including alcohol, was consumed
*__Activity__ (can be physical, cognitive or emotional) – please state what was the activity and for how long

| Enter time | Position/Activity | Blood Pressure | Heart Rate | Symptoms | Other details |
|---|---|---|---|---|---|
| **EARLY MORNING (ON WAKING)** Time: _ _ hr _ _min | | | | | |
| _ _ hr _ _ min | Lying | _ _ _/ _ _ _<br>sys. diast | | | |
| _ _ hr _ _min | After 3 min sitting | | | | |
| _ _ hr _ _min | After 3 min standing | | | | |
| **BREAKFAST** Time: _ _ hr _ _min; Details of food/fluid*: | | | | | |
| _ _ hr _ _ min | Lying | | | | |
| _ _ hr _ _ min | After 3 min standing | | | | |
| **ACTIVITY** Time: _ _ hr _ _min; Details of activity*: | | | | | |
| _ _ hr _ _ min | Before activity | | | | |
| _ _ hr _ _ min | After 3 min activity | | | | |
| **LUNCH** Time: _ _ hr _ _min; Details of food/fluid*: | | | | | |
| _ _ hr _ _ min | Lying | | | | |
| _ _ hr _ _ min | After 3 min standing | | | | |
| **ACTIVITY** Time: _ _ hr _ _min; Details of activity*: | | | | | |
| _ _ hr _ _ min | Before activity | | | | |
| _ _ hr _ _ min | After 3 min activity | | | | |
| **DINNER** Time: _ _ hr _ _min; Details of food/fluid *: | | | | | |
| _ _ hr _ _ min | Lying | | | | |
| _ _ hr _ _ min | After 3 min standing | | | | |



For peer review only - http://bmjopen.bmj.com/site/about/guidelines.xhtml

| **BEFORE SLEEPING (IN BED)** Time: _ _ hr _ _min | | | | | |
|---|---|---|---|---|---|
| 22.15pm **(In bed)** | Lying in usual sleeping position (as with pillows) | | | | |

**Measure sitting BP/HR only if you find it difficult to stand.**

**Please record any other type of activity that you would like to tell us about and is not listed above, with time & position.**

| **Enter time** | **Position/Activity** | **Blood Pressure** | **Heart Rate** | **Symptoms** | **Other details** |
|---|---|---|---|---|---|
| _ _ hr _ _ min | | | | | |
| _ _ hr _ _ min | | | | | |

For peer review only



For peer review only - http://bmjopen.bmj.com/site/about/guidelines.xhtml

# Adapted Autonomic Profile (aAP) protocol

### What does it entail?

Measuring blood pressure (BP) and heart rate (HR) at key times as outlined below while at home, with a personal approved home BP/HR monitor. An example is Omron, approved by the British Hypertension Society. The recordings provide information on how your autonomic nervous system responds to key activities in daily life such as postural change, before/after food and exertion. Experience over the decades indicates that it provides adequate data for initial diagnosis and for guidance on treatment.

Please record time, position, BP and HR, and key symptoms (such as dizziness) in brief on the accompanying **aAP diary sheet**. This is of particular importance in autonomic conditions and differs substantially from BP/HR recordings commonly used for high BP. Recordings should be taken on waking, after meals, after exertion and before sleep as outlined below:

- **WAKING** - Take a measurement lying, then after 3 minutes of sitting, then after 3 minutes of standing.
- **EATING** - After a standard meal (breakfast, lunch or dinner), within 10-15 minutes, take a measurement lying, then after 3 minutes of standing. Please note down what food and drink you have consumed (including alcohol) in the space provided.
- **EXERTION** - take a measurement After 3-5 mins of activity (physical, cognitive or emotional) morning and afternoon, separated from lunch and dinner. **NB:** exercise exertion levels will be different for everyone, an example of **physical exertion** can be 5 minutes of gentle walking, or up and down a flight of stairs. An example of **emotional exertion** might be watching an exciting sporting match or film. **Cognitive exertion** might be 5 minutes working out a crossword puzzle. We prefer everyone to attempt at least one form of physical exertion if possible. Please discuss with the clinical team what form of exercise or exertion may be most appropriate for you.

Note that the only reading which we would like you to do seated is the waking reading – a measurement initially on lying, then at sitting for 3 minutes and then at standing for 3 minutes. If it is difficult to stand at other times substitute sitting for standing, especially if after exertion or food.

If you wish to add additional activities, which worsens your symptoms, record them with the time, event/activity and position (lying, sitting, standing).

### Does it involve preparation?

Ensure that you choose a day when you can complete all of the measurements on the record sheet. It is intended to provide relevant autonomic information during a standard day with usual activities, and thus no change in schedule is needed. The aAP can be repeated on another day if needed.



For peer review only - http://bmjopen.bmj.com/site/about/guidelines.xhtml

**What are the advantages of doing the test ?**

The test will inform the clinician about the response of your autonomic system to some of the common triggers or stimuli in daily life. The test also helps you understand what makes your autonomic symptoms worse, which might help you modify some of these activities or triggers.

**Are there any risks of doing the test?**

There is a chance that standing may cause dizziness or even fainting for some people, so please ensure you are leaning against a wall when checking BP/HR on standing. If possible, have another person present in the room whilst performing the test standing. Abandon the test and sit or lie down if symptoms are worse.

**Does it cause discomfort, and are there after-effects?**

The BP cuff may feel uncomfortably tight for a short period if you have a high BP, as some may do, especially while lying down. There should be no after-effects.

**Where does it take place?**

The test can be undertaken in your own home and independent of the GP surgery or hospital. This avoids travel and can be performed whenever convenient. And it can be repeated to determine the effects of treatment.

**How is the result/event sheet forwarded?**

Please enter the results along with your name/ number and the date in the diary sheet and email or post to the clinician/ service:

Address

***The BP/HR autonomic profile and protocol originally was devised and evaluated for autonomic conditions by Professor Mathias, when he directed and developed the UK National Autonomic Referral Units, at St Mary's Hospital & the National Hospital for Neurology & Neurosurgery @ Queen Square in London. It has been adapted for home use in this protocol and has been of value in the Coronavirus era and its aftermath.***

**June 2022**



For peer review only - http://bmjopen.bmj.com/site/about/guidelines.xhtml

For peer review only
